# Colloidal Synthesis of Nickel Arsenide Nanocrystals for Electrochemical Water Splitting

**DOI:** 10.1021/acsaem.2c02698

**Published:** 2022-12-23

**Authors:** Fulvio Bellato, Michele Ferri, Abinaya Annamalai, Mirko Prato, Luca Leoncino, Rosaria Brescia, Luca De Trizio, Liberato Manna

**Affiliations:** † Istituto Italiano di Tecnologia (IIT), Via Morego 30, Genova16163, Italy; ‡ Università degli studi di Genova (UniGe), Via Dodecaneso 31, Genova16146, Italy

**Keywords:** transition metal arsenides, colloidal synthesis, electrocatalysis, hydrogen evolution reaction, oxygen evolution reaction

## Abstract

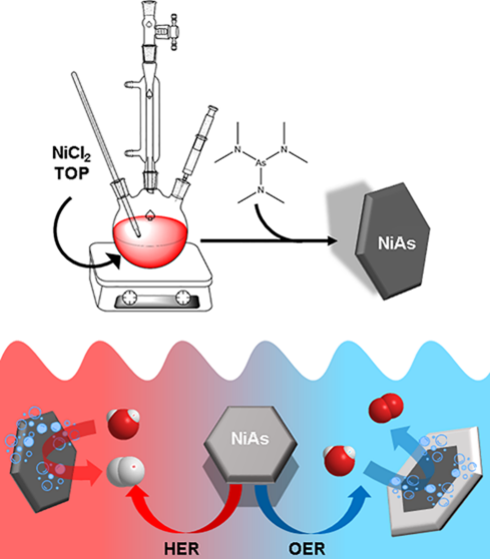

We report a detailed study on the first colloidal synthesis of NiAs nanocrystals. By optimizing the synthesis parameters, we were able to obtain trioctylphosphine-capped NiAs nanoplatelets with an average diameter of ∼10 nm and a thickness of *ca.* 4 nm. We then studied the performance of such NiAs nanocrystals as electrocatalysts for electrochemical water splitting reactions, namely, acidic hydrogen evolution reaction (HER) and alkaline oxygen evolution reaction (OER). These nanocrystals were found to be the most HER active ones among the transition metal arsenides reported to date despite exhibiting less than 40 h of stability under benchmark operative conditions (i.e., −10 mA cm_geo_^–2^). When tested as alkaline OER electrocatalysts, our NiAs nanocrystals behaved as a pre-catalyst and transformed superficially into an active Ni-oxy/hydroxide. As a result, NiAs nanocrystals featured an OER activity higher than that of benchmark Ni^0^ nanocrystals. Noticeably, the OER performance, in terms of , was retained for up to 60 h of continuous operation. The present study highlights how transition metal arsenides, whose structural features could be successfully controlled through a proper tuning of the synthetic parameters, might represent an emerging class of materials for electrocatalytic applications.

## Introduction

**Scheme 1:**
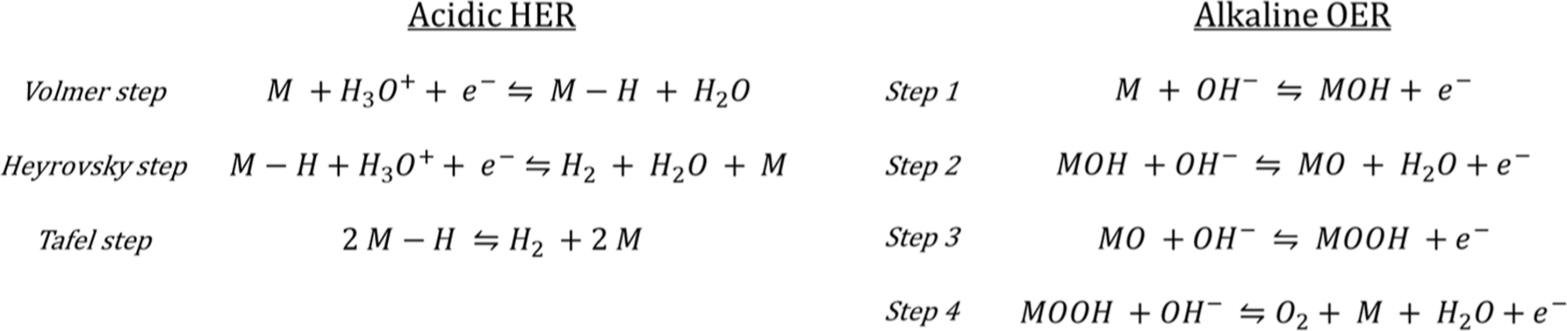
Acidic HER (Left) and Alkaline OER (Right) Model Reaction Pathways M represents a generic catalytic active site.

In a world that is facing the challenge of climate change and is therefore moving toward a greener future, H_2_ stands out as the most promising energy vector to replace fossil fuels.^1^ However, currently, *ca.* 95% of the H_2_ produced worldwide is classified as blue or gray H_2_,^2^ that is, it originates from fossil fuel-based processes such as the steam methane reforming which causes the production of massive volumes of CO_2_.^2^ Green hydrogen, obtained by electrochemical water splitting, addresses this problem, guaranteeing the CO_2_-free production of H_2_ when coupling the process (i.e., powering the electrolyzer) with electricity generated from renewable sources.^1^ The electrochemical water splitting process consists of two distinct reactions: the hydrogen evolution reaction (HER), taking place at the cathode, and the oxygen evolution reaction (OER), at the anode. Scheme 1 summarizes the basic reaction pathways of HER and OER under acidic and alkaline pH, respectively, which are the ideal conditions for the two separate reactions^3^ (i.e., they do not involve water dissociation as the first step).

From the early studies of electrochemical water splitting, platinum group metals (PGMs) have demonstrated unmatched activities in both HER and OER, yet these metals have limited availability and high cost.^1,3^ In the last 2 decades, transition metal (TM) chalcogenides and pnictogenides have been extensively investigated as low-cost and efficient alternatives to PGM-based electrocatalysts for acidic HER,^4,5^ and other energy-related electrocatalytic processes (e.g., electrochemical CO_2_ and N_2_ reduction).^6^ Similarly, TM compounds have also been tested for alkaline OER,^7^ in this case with the aim of overcoming the activity limitations of state-of-art catalysts such as Raney Ni.^8^ The promising performance shown by these materials in both anodic and cathodic processes is generally attributed to the peculiar bonding behavior of TM-metalloids compounds and their catalytic dual-site nature (i.e., possible participation of both TM and metalloids atoms to the actual electron transfer).^9,10^

TM phosphides are among the most investigated alternatives to PGM-based catalysts.^5^ In particular, nickel phosphide (Ni_2_P) exhibited promising performance when tested for acidic HER,^11^ while Ni-based catalysts are currently the state-of-the-art ones for alkaline OER.^1^ Despite arsenic lying in the metalloids region of the periodic table, right under phosphorous, only a few reports on TM arsenides (TMAs) for electrocatalytic applications can be found in the literature,^10,12^ with NiAs (synthesized in the form of nanostructured thin films) being reported as a promising OER catalyst under alkaline conditions only by Masa et al.^10^ As opposed to TM phosphides, the substantial lack of studies on TMAs at the nanoscale level does not allow us to assess their potential in practical applications. A major hurdle in the exploration of the electrocatalytic features of TMA nanocrystals (NCs) is represented by the limited colloidal synthesis routes developed to date for metal arsenide NC compounds, with InAs and GaAs representing exceptions.^13^ This is mainly due to the poor availability of As precursors with the most used ones being tris(trimethylsilyl)arsine or tris(trimethylgermanyl)arsine,^14^ which are toxic, pyrophoric, difficult to handle, and their availability on the market is very limited.^15^ As NiAs deserves a closer attention in virtue its interesting features,^10^ developing a synthetic route for NiAs NCs should unravel its actual potential as a water splitting catalyst; following the path traced by the reports on InAs NC synthesis, the use of alternative, cheaper, and less toxic arsenic precursors [e.g., tris(dimethylamino)arsine (amino-As)]^13^ has been explored.

In this study, we report the first amino-As based colloidal synthesis of NiAs NCs which feature a hexagonal platelet shape with a diameter of *ca.* 10 nm and *ca.* 4 nm thickness, coated with trioctylphosphine (TOP). We have also studied in detail the catalytic properties of these NCs when employed as water splitting electrocatalysts, namely, in the acidic HER and alkaline OER processes. A further preliminary screening of NiAs HER performances under alkaline pH was carried out. The NCs are compared with the state-of-art electrochemical water splitting catalysts through electrochemical metrics (i.e. onset potential, Tafel slope, overpotential at benchmark current densities). Based on them, we demonstrate that our NiAs NCs outperform other TMAs and Ni^0^ NCs in terms of HER and OER intrinsic activity, hence highlighting that metal arsenides deserve further investigation on their possible catalytic applications.

## Experimental Section

### Synthesis of NiAs NCs

In a 25 mL flask, a solution containing 0.4 mmol of NiCl_2_ (98%, purchased form Sigma-Aldrich), 6 mL of 1-octadecene (ODE, 97%, purchased from Sigma-Aldrich), and 3 mL of TOP (97%, purchased from Strem Chemicals) was degassed for 2 h at 120 °C under vacuum using a standard Schlenk line. Upon complete degassing, the atmosphere was switched to argon (Ar) and the solution was heated up to 250 °C. A solution containing 0.4 mmol of tris(dimethylamino)arsine (99%, purchased from Strem Chemicals) in 0.5 mL of TOP, prepared under inert conditions (i.e., in glovebox), was then swiftly injected into the reaction flask. The reaction time was set to 1 min. Then, the solution was cooled down to room temperature. The NiAs NCs obtained in the synthesis were washed 3 times with a mixture of anhydrous toluene (99.8%, purchased from Sigma-Aldrich) and anhydrous ethanol (99.9%, purchased from Carlo Erba Reagents), each rinsing followed by centrifugation (4500 rpm, 10 min) and removal of the supernatant. Finally, NiAs NCs were dispersed in toluene or hexane and stored in a glovebox.

### Synthesis of Ni^0^ NCs

Ni^0^ NCs were synthesized following the procedure reported by Carenco et al.^16^ In a 25 mL flask, 1 equiv of nickel acetylacetonate [Ni(acac)_2_, 95%, purchased by Sigma-Aldrich] and 10 equiv of OLAM were mixed and degassed in a standard Schlenk line under vacuum for 2 h at 100 °C. Afterward, the atmosphere was switched to argon and the solution was heated up to 220 °C. The reaction time was 2 h. Then, the solution was cooled down to room temperature. Ni^0^ NCs were washed three times with a mixture of hexane (99%, purchased from Sigma-Aldrich) and ethanol (99.9%, purchased from Carlo Erba Reagents), each rinsing followed by centrifugation (4500 rpm, 10 min) and discarding of the supernatant. The Ni^0^ NCs were dispersed in hexane for further characterizations/experiments.

### Ligand Stripping Procedure

The ligand stripping treatment was carried out according to the procedure reported by Liu et al.^17^ with only slight modifications. In brief, a stripping solution was prepared by dissolving 4 mmol of PbI_2_ (99.999%, purchased from Sigma-Aldrich) and 1.6 mmol of ammonium acetate (99%, purchased from Sigma-Aldrich) in 40 mL of dimethyl-formamide (DMF, 99.8%, purchased from Sigma-Aldrich). Then, 5 mL of NiAs NCs (4.5 mg/mL dispersion in hexane) were mixed with 5 mL of the stripping solution. The mixture was vigorously shaken for 1 min; as a consequence of the successful ligand stripping, NiAs NCs transferred to the DMF phase (Figure S6). NiAs NCs were washed three times with toluene, to completely remove stripped ligands, and once with DMF, to remove any trace of PbI_2_ and ammonium acetate. NiAs NCs were then dried under vacuum at 80 °C for 2 h; the final ligand-stripped NiAs NC powder was then used to prepare catalytic inks.

### Characterization

#### Scanning Electron Microscopy Analyses

Before imaging, specimens were prepared by spreading few milligrams of powder onto a carbon tape loaded on an aluminum stub; then, a conductive carbon coating (*ca.* 10 nm thick) was sputtered using a high-resolution sputter coater (Emitech K950X). The samples were studied on a field emission scanning electron microscopy (FE-SEM) JSM-7500 FA (JEOL) and a SEM JSM-6490 (JEOL). Both microscopes were operated at 10 kV acceleration voltage, for imaging, and considering back-scattered electrons for enhancing differences in chemical composition. In order to detect and quantify the elements of interest, both microscopes were operated at 25 kV and energy dispersive X-ray spectroscopy (EDS) analyses were performed using, respectively, a detector X-Max [80 mm^2^ area, silicon drift detector (SDD), Oxford Instruments] and a detector JED-2300 EX-54165JNH (10 mm^2^ area, Si/Li detector, JEOL).

#### TEM Analyses

**Figure 1:**
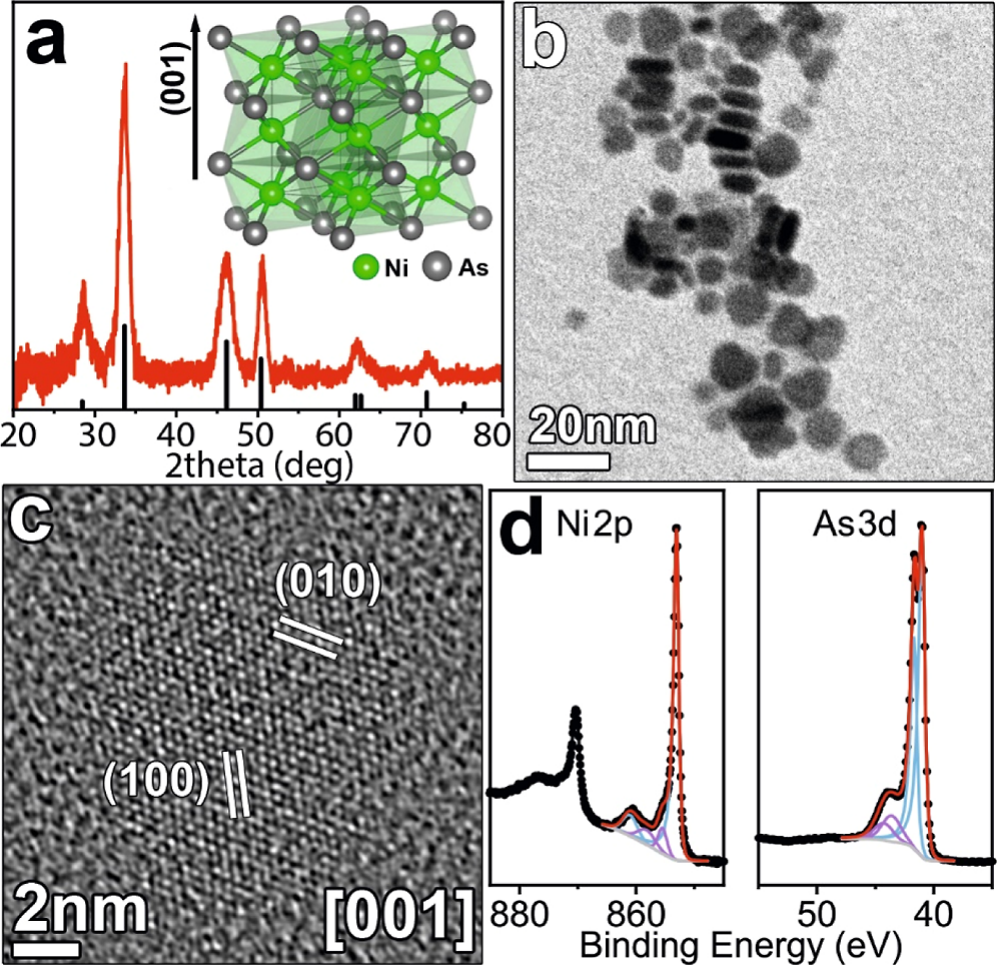
(a) NiAs NCs XRD pattern compared with the powder diffraction file for the database structure ICDS 611040. In the inset, the schematic model of the NiAs crystal structure;^20^ (b) BF-TEM image of NiAs NCs and (c) HRTEM image of a selected NiAs NC, indexed based on hexagonal NiAs (ICSD 611040). (d) High-resolution XPS spectra of the Ni 2p and As 3d regions.

Transmission electron microscopy (TEM) specimens were prepared by drop-casting a NC dispersion in isopropyl alcohol onto carbon-coated copper grids. Bright field TEM (BF-TEM) analyses were performed on a JEOL JEM-1011 TEM transmission electron microscope operating at 100 kV. High angle annular dark field scanning TEM (HAADF-STEM) and high-resolution TEM (HRTEM) analyses were carried out on an image-*C*_s_-corrected JEOL JEM-2200FS microscope (Schottky emitter) operated at 200 kV and equipped with a Bruker XFlash-5060 SDD-based EDS system. The STEM–EDS maps were obtained by integration of the Kα peaks for C, O, Ni, and As. For HRTEM imaging, in order to reduce carbon contamination, the NCs were exposed to a low dose rate [∼30 electrons/(Å^2^ s)] and HRTEM images were acquired using a direct electron detection camera (K2 Summit, Gatan), in the super-resolution mode. The HRTEM images shown here (Figure 1c) were obtained from a (270 nm)^2^ frame composed by summing aligned frames acquired by short exposure (0.3 s), with a total acquisition time of 12 s.

#### X-ray Diffraction

X-ray diffraction (XRD) measurement were carried out on a PANalytical Empyrean diffractometer operating at 45 kV and 40 mA, equipped with a 1.8 kW Cu Kα ceramic X-ray tube and a PIXcel^3D^ 2 × 2 area detector.

#### X-ray Fluorescence Spectroscopy

X-ray fluorescence (XRF) measurement were performed on a Bruker M4 TORNADO micro XRF equipped with 30 W micro-focus Rh polycapillarity lens (spot size < 25 μm for Mo Kα), and the signal was detected by 2 × 30 mm^2^ energy-dispersive X-ray SDDs, with energy resolution <145 eV (for Mo Kα).

#### X-ray Photoelectron Spectroscopy

X-ray photoelectron spectroscopy (XPS) analyses were carried out on a Kratos Axis UltraDLD spectrometer using a Mg Kα source operated at 20 mA and 15 kV. Survey scan analyses were carried out on an analysis area of 300 × 700 μm and a pass energy of 160 eV. High-resolution analyses were carried out over the same analysis area at a pass energy of 20 eV. The spectra were charge corrected to the main line of the carbon 1s spectrum (adventitious carbon) set to 284.8 eV. The spectra were analyzed using the CasaXPS software (version 2.3.25).

#### ICP-OES Analysis

A known amount of NiAs NCs were dissolved in a HCl/HNO_3_ (3:1, V/V) mixture. The obtained solution underwent quantitative elemental analysis via inductively coupled plasma optical emission spectroscopy (ICP-OES) using an iCAP 7600 DUO Thermofisher Scientific spectrometer.

### Preparation of Electrodes

Ligand stripped NiAs NCs were dispersed in a water/isopropanol mixture in order to obtain a catalytic ink. Briefly, 3.8 mg of stripped NiAs NCs were dispersed in 0.3 mL (*ca.* 12.5 mg_NiAs_ mL^–1^) of a 1:1 (volumetric ratio) Milli-Q water (18.2 Ω, Millipore) to isopropanol (ACS reagent, ≥99.5%, purchased from Sigma-Aldrich). *ca.* 25 μL of a Nafion 117 solution (∼5% in a mixture of lower aliphatic alcohols and water, purchased from Sigma-Aldrich) was then added to the suspension to achieve a Nafion/NiAs mass ratio equal to *ca.* 0.3. The dispersion was sonicated for 30 min to yield a homogeneous ink. 5 μL of the catalytic ink was drop-cast on a 0.5 × 0.5 cm plasma treated H090 Toray paper. The final NiAs/Toray paper electrodes had a nominal loading of *ca.* 0.25 mg_NiAs_ cm^–2^. The electrodes were dried on a hotplate at 50 °C for 30 min before use.

### Electrochemical Testing

Both HER and OER electrochemical tests were performed in a three-electrode configuration using NiAs/Toray paper electrodes as working electrodes (WEs) and a leakless Ag/AgCl (3 M KCl, purchased from eDAQ) as reference electrode. Two different counter electrodes were used, namely, a carbon graphite rod (150 mm, *ø* 3 mm, low density, 99.995% trace metals basis, purchased from Sigma-Aldrich) or a Pt wire for HER and OER testing, respectively. 0.5 M H_2_SO_4_ and 1 M KOH aqueous solutions were used as electrolytes for acidic HER and alkaline OER testing, respectively. Both solutions were treated with Chelex 100 resin (Bio-Rad, catalog no. 210011676) to avoid any possible metal contamination. The electrolytes’ pH was measured with a SensION+ PH3 pH meter equipped with a Crison glass electrode (0 to 14 pH range). pH values of 0.41 (0.5 M H_2_SO_4_) and 13.81 (1 M KOH) were measured.

Electrochemical tests were carried out on an Ivium CompactStat-e potentiostat. For both HER and OER, we followed a protocol that included several electrochemical techniques: cyclic voltammetry (CV), potentiostatic electrochemical impendence spectroscopy (PEIS), linear sweep voltammetry (LSV), chronoamperometry (CA), and chronopotentiometry (CP). All the experiments were repeated at least three times on as many fresh NiAs/Toray paper electrodes. Full details about the electrochemical characterization are available in the Supporting Information. Unless differently stated, all potentials reported were converted into the RHE scale and IR corrected according to the following equation
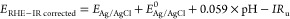
where *R*_u_ is the uncompensated system resistance determined by PEIS (details in the Supporting Information, Figures S12 and S29 for typical Nyquist plot collected under acidic HER and alkaline OER conditions, respectively).

After having undergone electrocatalytic tests, the used electrodes were rinsed with Milli-Q water, dried under N_2_ flow at room temperature and characterized as reported in the dedicated section.

## Results and Discussion

### Synthesis and Characterization of NiAs NCs

We developed a colloidal synthesis approach capable of delivering NiAs NCs that are coated with TOP. In a typical synthesis, NiCl_2_ is dissolved in ODE and TOP at 120 °C, heated up to 250 °C and, eventually, amino-As is injected in the reaction flask to trigger the NCs’ nucleation and growth. The detailed procedure and the optimization of the reaction parameters are described in the Experimental Section and Supporting Information, respectively (Figures S1–S3 and Table S1). The XRD pattern of the product indicates that the NCs have a hexagonal close-packed crystal structure (Figure 1a, peaks assignment in Table S2), belonging to the *P*6_3_/*mmc* space group (ICSD 611040). In this structure, Ni atoms occupy octahedral sites formed by hexagonally close packed As atoms. Such As atoms are in turn surrounded by six Ni atoms according to a prismatic triangular coordination (inset in Figures 1a and S4a). Interestingly, in such a crystal structure, Ni atoms form columns along the (001) direction, therefore promoting a metallic character (*i.e.* electrical conductivity) through the formation of Ni–Ni bonds (Figure S4b).^18^ BF-TEM images (Figure 1a) of NiAs NCs evidenced that the use of TOP yielded disk-like NCs with an average diameter and thickness of *ca.* 10 and 4 nm, respectively (Figures 1b and S4c). The elemental analyses, performed via ICP-OES (Table S3) and XRF (Figure S5) indicated a Ni/As ratio of 1:1 in the samples. HRTEM images (Figure 1c), revealed that NiAs NCs are monocrystalline disks, with the extended facets parallel to {001} planes. Eventually, XPS analyses (Figure 1d) were fully consistent with previous reports on NiAs,^19^ with the asymmetric Ni 2p_3/2_ peak located at 853.1 ± 0.2 eV and a satellite at 861.1 ± 0.2 eV, and the asymmetric As 3d_5/2_ peak centered at 41.0 ± 0.2 eV (light blue profiles in Figure 1d). XPS also evidenced traces of surface oxidation (purple profiles in Figure 1d) on both Ni [peaks at *ca.* 855.5 and 858 eV, in a range usual for NiO and Ni(OH)_2_] and As signals (peaks at *ca.* 43.6 and 44.3 eV), possibly due to air exposure before the analyses. The Ni/As atomic ratio, as obtained through XPS quantitative analysis, was 0.8:1. The discrepancy with the 1:1 ratio emerged with other techniques was ascribed to the presence of the said oxidation and to the surface sensitivity of XPS.

Finally, it is worth mentioning that pure phase NiAs NCs could be synthesized also by employing oleylamine as the sole surfactant (Table S1), although with a lower control over size and morphology compared to TOP (Figure S3e,f). Therefore, only TOP-synthesized NiAs NCs were further investigated here. Interestingly, no reducing agent was employed to prepare NiAs NCs with amino-As, differently from what observed for the synthesis of InAs NCs performed at similar temperatures (e.g., InAs NCs based on amino-As).^13^ Indeed, As^3+^ present in amino-As has to be reduced to As^3–^ in order to form the metal arsenide NCs.^13,21^ We hypothesize that NiCl_2_ might be capable of promoting such a reduction, most likely by favoring the 4As^3+^ → 3As^5+^ + As^3–^ disproportionation reaction, in analogy with what observed for ZnCl_2_ toward amino-P.^22,23^

### Fabrication and Characterization of the NiAs Electrodes

Before testing their electrochemical activity, the NiAs NCs were subjected to a ligand stripping procedure with a solution of PbI_2_ and CH_3_COONH_4_ in DMF^17^ as detailed in the Experimental Section. The effectiveness of the ligand stripping procedure was confirmed by the evident transfer of NiAs NCs from the hexane to the more polar DMF phase (Figure S6a). The retention of the starting crystal phase and morphology after the ligand stripping procedure were assessed by XRD and TEM (Figure S6b). The stripped NiAs NCs were then used to prepare electrodes (Experimental Section for details). The substrate chosen was carbon paper (H090 Toray paper), which was priorly plasma-treated to impart the necessary hydrophilicity for the homogeneous spreading of the NiAs NC catalytic inks. The choice of a porous 3D carbonaceous structure over the typical flat glassy carbon was dictated by (i) the larger area of the support itself, allowing for a better dispersion for unsupported NCs^24^ and (ii) the easier post-mortem characterization.

**Figure 2:**
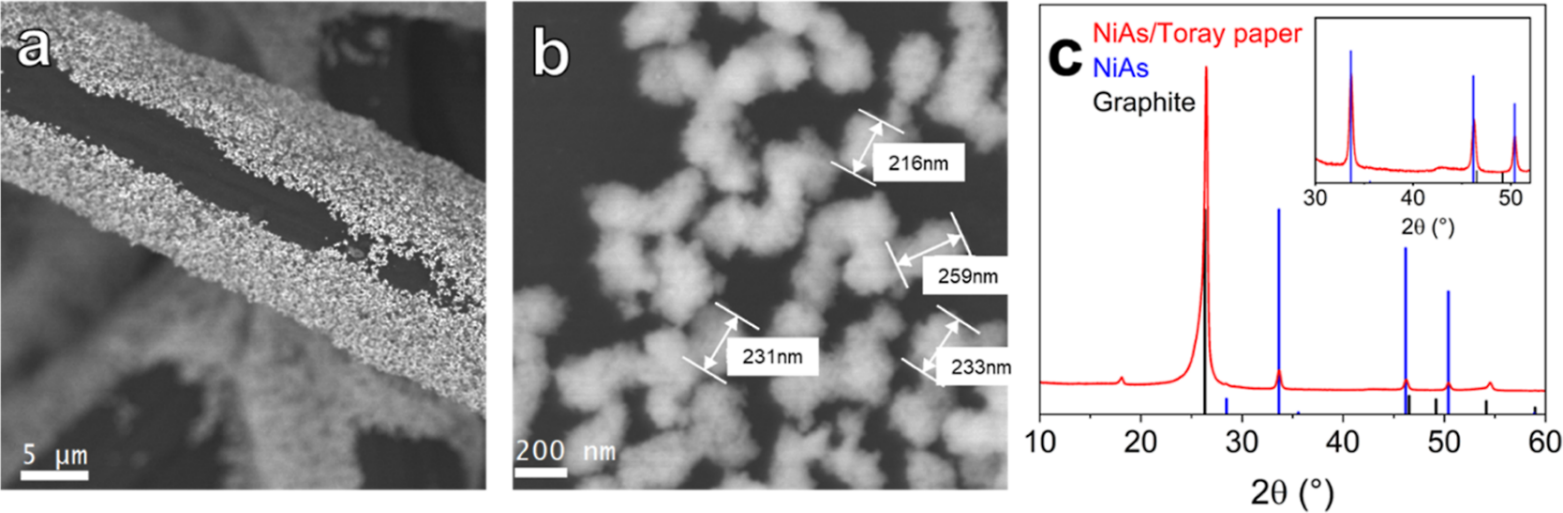
(a,b) FE-SEM micrographs of pristine NiAs/Toray paper electrodes. (c) Related XRD pattern; blue and black bars represent reference reflections of bulk NiAs (hexagonal, ICSD 611040) and graphite (ICSD 76767), respectively. In the inset: magnification of the 30–50° 2θ range where NiAs main peaks are located.

FE-SEM images of the drop-cast NiAs/Toray paper electrodes (mass loading equal to *ca.* 0.25 mg_NiAs_ cm^–2^) evidenced a homogeneous distribution of the active phase over the whole substrate (Figure 2a). At higher magnification (Figure 2b), it is possible to notice how the original NiAs NCs had partially agglomerated onto the substrate, forming aggregates with a size of *ca.* 200 nm.

The XRD pattern collected on NiAs/Toray paper electrodes displayed the typical NiAs reflections (Table S2) along with the reflections of graphite (main peak at 26.4°), attributed to the support (Figures 2c and S7). This confirms that the drop-casting procedure (and all the related processing of NiAs NCs) does not affect the phase purity of the samples, apart from promoting agglomeration. The homogeneous dispersion of the active phase onto the support and the retention of the former NiAs stoichiometry were further corroborated by SEM-coupled EDS (SEM–EDS) and XRF spectra/maps (Figures S8 and S9 and Table S4).

### Hydrogen Evolution Reaction

**Figure 3:**
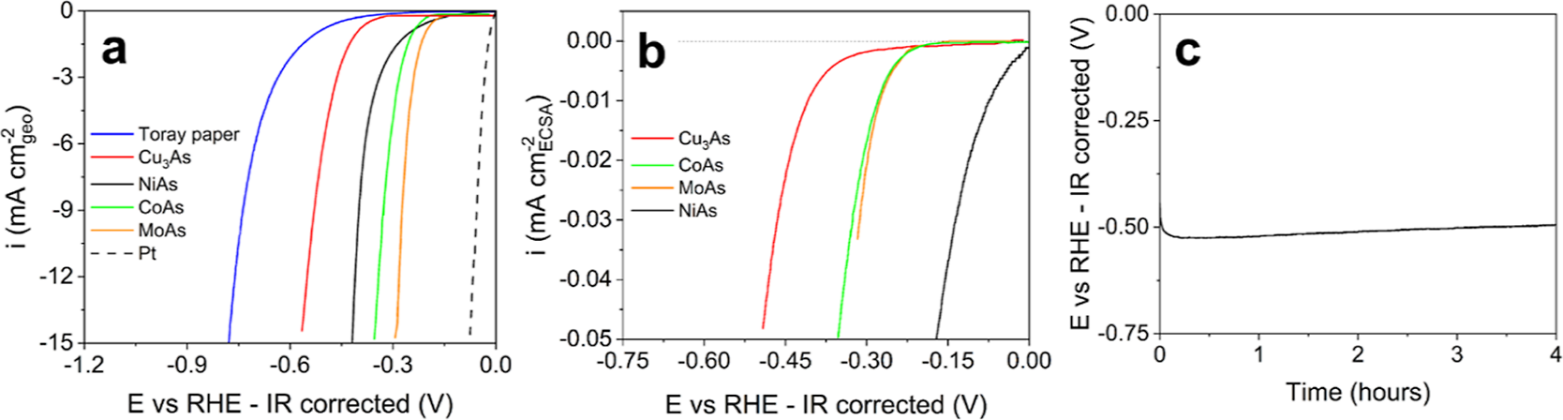
Evaluation of the electrochemical HER activity of NiAs NCs. LSVs (scan rate = 2 mV s^–1^) of NiAs, previously reported TMAs^12^ and benchmark Pt nanoparticles, displayed as potential vs (a) geometrical and (b) ECSA-corrected current densities. (c) 4 h long chronopotentiometric measurement at −10 mA cm_geo_^–2^.

The electrocatalytic performance of NiAs NCs toward HER was tested under acidic conditions (0.5 M H_2_SO_4_) in a typical three-electrode configuration. All experimental details are discussed in the dedicated section and in the Supporting Information (Section 1.3 and Table S5). After the electrode conditioning (i.e., continuously cycling the potential in the HER window, until convergent curves were obtained, Figure S10), a first investigation of the HER activity of NiAs NCs was carried out by recording LSV curves. The NiAs NCs exhibit an onset potential (*E*_onset_, defined as the potential at which current reaches −1 mA cm_geo_^–2^) of *ca.* 250 mV and reach a geometrical current density of −10 mA cm_geo_^–2^ at a *ca.* 400 mV overpotential (Figure 3a), outperforming the previously reported Cu_3_As,^12^ but apparently being less active than both CoAs and MoAs (Table 1). All TMAs are however less active than Pt (platinum nanopowder, <50 nm by TEM, purchased from Sigma-Aldrich. Electrodes prepared with the same procedure, loading and Nafion/active phase ratio reported for NiAs/Toray paper electrodes). However, a proper comparison of the intrinsic activity of the electrocatalysts cannot be performed based on geometrical current densities, which are well known to depend on several parameters such as the porosity of electrodes and mass loading of the active phase.^25^ Normalization of the delivered current by the electrochemically active surface area (ECSA) allows a fair comparison of the electrocatalytic activity of different materials. The rather limited literature on TMAs generally reports ECSA-corrected data by applying the double-layer capacitance (*C*_dl_) method;^12^ hence, we followed the same approach for our samples. ECSA-corrected LSVs of NiAs and previously reported TMAs are displayed in Figure 3b, while the procedure for ECSA determination^26^ and related calculations are detailed in the Supporting Information (Figure S13 and Table S6). The HER normalized activity of NiAs NCs was higher than that of previously reported TMAs, reaching a −0.03 mA cm_ECSA_^–2^ at an overpotential of 136 mV (Table 1).

**Table 1:** Key Electrochemical HER Parameters Derived from LSV (*E*_onset_) and Chronopotentiometric Scans (η^HER^) for NiAs and Other TMAs^a^

	*E*_onset_^HER^^b^ (mV)	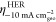 (mV)	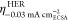 (mV)	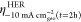 (mV)
NiAs (this work)	252	400	136	510 (470^d^)
MoAs^c^	205	276	313	303
CoAs^c^	243	334	327	362
Cu_3_As^c^	406	536	464	543
Pt^e^	≈0	≈40		

a All potentials are negative, minus sign omitted.

b *E*_onset_ defined as the overpotential at which −1 mA cm_geo_^–2^ current densities are achieved.

c Samples obtained as nanostructured thin films.

d Data collected after 35 h of operation (not reported for other TMAs).

e Pt/Toray paper electrodes, prepared from a dispersion of commercial Pt nanoparticles (<50 nm), following the same procedure reported for NiAs/Toray paper ones.

From a kinetic and mechanistic point of view, the measurement of Tafel slopes allows one to investigate the current–voltage relationship for a specific catalyst and, therefore, to assess the rate-determining step of HER on the catalyst.^27^ The Tafel slope of NiAs NCs, obtained from LSVs, stands at around 92 mV dec^–1^ (Figure S14a), in line with the values reported for MoAs and CoAs (*ca.* 75 mV dec^–1^). These results indicate the Volmer step (Scheme 1, theoretical Tafel slope *ca.* 120 mV dec^–1^) as the most likely rate-determining step of HER on TMAs (Heyrovsky and Tafel steps exhibit theoretical Tafel slopes around 40 and 30 mV dec^–1^, respectively^27^). Nevertheless, it must be stated that the use of potentiodynamic techniques is not recommended for the determination of Tafel slopes, as the inevitable contribution of capacitive currents results in unreliable data.^28^ When regressing the Tafel slopes from a potentiostatic approach (Figure S14b), NiAs exhibits values higher than 120 mV dec^–1^ (*ca.* 245 mV dec^–1^), thus indicating a generally hindered HER process on NiAs NCs, differently from the more promising Ni_2_P features (Tafel slope of *ca.* 46 to 81 mV dec^–1^^11^ depending on the overpotential range). A more detailed discussion on HER Tafel slopes measurements is reported in the Supporting Information.

In order to benchmark the activity and stability of NiAs NCs, chronopotentiometric tests at −10 mA cm_geo_^–2^ were performed (Figure 3c). NiAs exhibits a  of *ca.* 500 mV at 2 h, in contrast with the lower values reported for other TMAs (Table 1), but consistent with the actual performance of NiAs in terms of *E* versus geometric current density (Figure 3a). For longer operational times, the recorded  fluctuates in the range from 420 to 520 mV; then, the catalyst loses its activity after *ca.* 40 h (Figure S15). Such a noisy electrochemical trace is possibly due to NiAs instability under acidic HER conditions. As a matter of fact, voltammetric curves, collected after 30 h long chronopotentiometric tests, consistently display a decrease in the NiAs HER performance (dotted curve in Figure S16), especially at large current densities. Post-mortem SEM images of NiAs electrodes confirm the leaching/dissolution of the active phase from the WE (Figure S17). XPS data consistently depict a surface on which NiAs NCs are almost absent (Figure S18), further confirming the poor stability of NiAs under acidic HER conditions. In turn, the instability of NiAs NCs under acidic HER conditions might be the cause of the >120 mV dec^–1^ Tafel slopes registered through the step-wise potentiostatic approach. Indeed, an accelerated leaching/dissolution of NiAs NCs might be envisaged when operating at larger current densities as in the case of the step-wise chronopotentiometric tests registered for Tafel slopes’ regression (Figure S14b, inset).

To complete the picture, NiAs HER performance under alkaline conditions was investigated as well; the reader is invited to refer to the Supporting Information, which reports the electrochemical data and physical–chemical characterizations (Section 1.4a, Figures S19–S25, and Tables S7 and S8). Briefly, NiAs HER performance under alkaline conditions outpaced the one registered at acidic pH, both in terms of electrocatalytic metrics ( = 225 mV) and stability (up to 60 h of operation, Figure S22). However, despite these interesting results, NiAs NCs do not offer a valid alternative to the cheap cathodes (e.g., perforated Ni-coated stainless steel electrodes^1^) already employed in alkaline HER. Anyways, a further investigation of TMAs HER performance at alkaline pH is undoubtedly of interest in the pursuit of more efficient and stable catalysts.

### Oxygen Evolution Reaction

NiAs/Toray paper electrodes were also tested for the OER under alkaline conditions (1 M KOH). With Ni^0^-based catalysts representing the benchmark for alkaline OER,^29^ assessing the OER performance of NiAs NCs in comparison with that of Ni^0^ NCs allows us to highlight the electrocatalytic features of the arsenide. For this purpose, Ni^0^ NCs were synthesized (details in the Experimental Section, characterizations reported in Figure S26) and processed similarly to NiAs NCs in order to prepare Ni^0^/Toray paper electrodes.

Prior to recording OER catalytic data, both NiAs/Toray paper and Ni^0^/Toray paper electrodes were conditioned by continuous potential cycling in a wide potential range, until convergent curves were obtained (Figure S27, further considerations on the CVs are available in the Supporting Information). As expected, in both curves, a faradaic peak around 1.4 to 1.5 V versus RHE was detected, accounting for the Ni(II) to Ni(III) oxidation, the latter being extensively reported in the literature to be the OER active site for Ni-based materials.^29^ During the potential cycling, it can be noted that such anodic peaks increase steadily until reaching a stable area after *ca.* 50 cycles (insets in Figure S27), most likely indicating the growth of a nickel oxide and/or hydroxide onto both NiAs and Ni^0^ NCs.^10,30^ Interestingly, the height and area of the Ni(II) to Ni(III) oxidation peak registered for Ni^0^ are more than twice those of the corresponding NiAs NCs sample, suggesting that Ni^0^ NCs develop a larger number of surface Ni(III) active sites.

**Figure 4:**
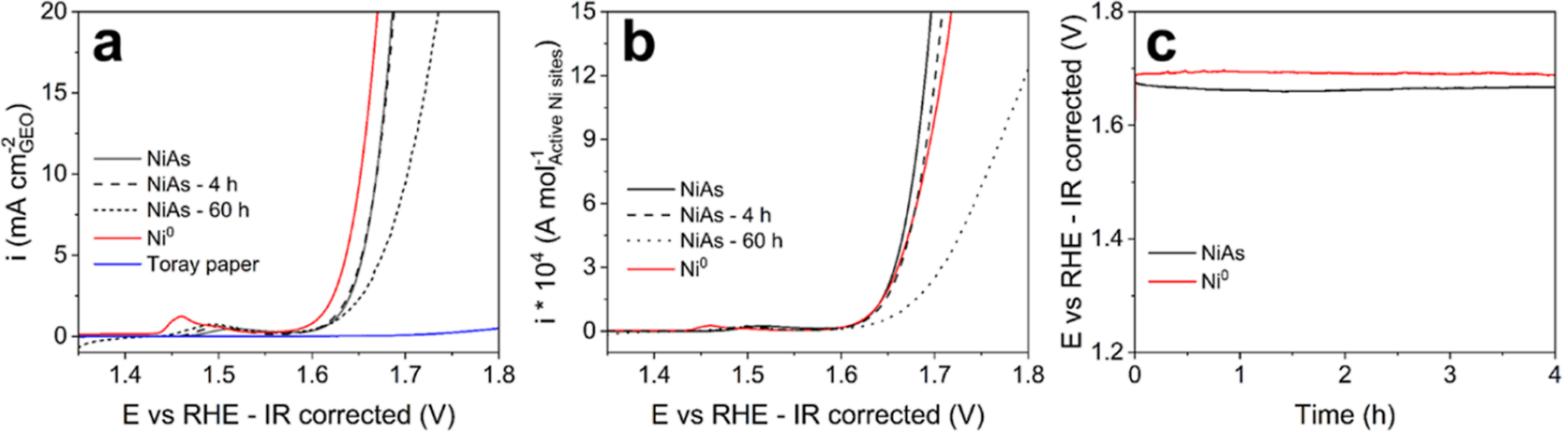
Evaluation of the electrochemical OER activity of NiAs. LSVs (scan rate = 2 mV s^–1^) of NiAs and Ni^0^ NCs displayed as potential vs (a) geometrical and (b) number of active sites-normalized current densities. (c) 4 h long chronopotentiometric measurement at 10 mA cm_geo_^–2^.

LSVs of NiAs NCs, Ni^0^, and bare Toray paper are displayed in Figure 4a. NiAs (black solid line) exhibits an OER onset potential of 1.613 V and delivers 10 mA cm_geo_^–2^ at an applied voltage of 1.669 V, resulting in a  of *ca.* 439 mV. On the other hand, the bare support (blue solid line) does not present any OER activity. Despite an inversion at *i* > 150 mA cm_geo_^–2^ (Figure S30a), Ni^0^ NCs appear to outperform NiAs NCs, both in terms of *E*_onset_ and  (Table 2). As discussed in the previous section, electrochemical parameters extracted from geometrical current-based data may not represent the intrinsic activity of an electrocatalyst and hinder a proper comparison of different materials. When testing Ni-based catalysts for OER applications, it is common practice to assess the ECSA in terms of number of active sites [i.e., moles of formed Ni(III) species] from the Ni(II) to Ni(III) area.^10,31,32^ The number of active Ni(III) sites for both NiAs and Ni^0^ electrodes was therefore evaluated by integrating the Ni(II) to Ni(III) peaks [Figure S31, further discussion on Ni(II) to Ni(III) oxidation are available in the Supporting Information]. The normalized voltammetric curves are reported in Figure 4b (and Figure S30b). Such normalization evidences that the intrinsic catalytic activity of NiAs NCs is comparable to that of Ni^0^ in the low overpotential region, while under higher applied potentials (i.e., larger current densities), NiAs outperforms Ni^0^ NCs, more than doubling the delivered current at *E* > 1.8 V versus RHE. Accordingly, the catalytic turnover frequency of NiAs deviates from that of Ni^0^ as the applied overpotential increases (Figure S32 and Table S9). The reason for the inversion in the activity trend with respect to the one observed in Figure 4a has to be sought in the larger number of active Ni(III) sites exhibited by Ni^0^ NCs.

**Table 2:** Key Electrochemical OER Parameters Derived from (a) LSV and (b) Chronopotentiometric Scans for NiAs NCs and Ni^0^ NCs

	*E*_onset_^OER^^a^ (V)	a (V)	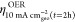 (V)
NiAs	1.613	0.439	0.431 (0.467^b^)
Ni^0^	1.605	0.422	0.462

a *E*_onset_ defined as the overpotential at which −1 mA cm_geo_^–2^ current densities are achieved.

b Data collected after 50 h of operation.

In terms of kinetic parameters, literature reports OER Tafel slopes for Ni-based catalysts ranging from 50–60 to 120 mV dec^–1^.^27^ Regressing the Tafel slope of NiAs NCs from potentiostatic data (Figure S33b), a dependence on the overpotential, as often reported for OER catalysts,^33−35^ can be spotted. For η < 0.55 V, NiAs exhibits a Tafel slope of *ca.* 120 mV dec^–1^; according to microkinetic analyses and theoretical models, such a slope value is observed for large coverages of surface species formed in the step previous to the rate-determining one.^27^ Therefore, the single Tafel slope is in this case not sufficient to unveil the exact step representing the bottleneck in the OER mechanism onto NiAs. In the high overpotential region (η > 0.55 V), a steeper value of the Tafel slope is obtained (*ca.* 225 mV dec^–1^). Although already documented for Pt in 1 M KOH,^35^ the almost doubling of the Tafel slope with the increase in potential is difficult to be addressed as it could stem from both surface coverage-related issue or simply from diffusional limitations. A more detailed discussion on OER Tafel slopes measurements and results is reported in the Supporting Information.

Chronopotentiometric tests, presented in Figure 4c (and Figure S30c), were collected to assess both  and stability of NiAs. Apart from a 2 s long initial transition period during which Ni^0^ NCs display lower OER overpotential (Figure S34), NiAs exhibits a  equal to 431 mV, *ca.* 30 mV lower than Ni^0^ NCs (Table 2). Moreover, NiAs/Toray paper electrodes demonstrated robust performance, delivering a 10 mA cm_geo_^–2^ current density for more than 60 h with only a slight increase in the overpotential (*ca.* 25 mV after 60 h of operation, Figure S30c). LSVs collected on after 4 h-long chronopotentiometric (10 mA cm_geo_^–2^) tests are identical to those registered on pristine NiAs/Toray paper electrodes (dashed black line in Figures 4a and S30a). However, after 60 h of operation, the voltammetric curve shows a major decrease in performance, which is more evident at higher applied potentials (dotted black line in Figures 4a and S30a). It is noteworthy that the decrease in the intrinsic activity of NiAs is even more pronounced (Figures 4b and S30b) when considering that during operation the number of active sites is increasing (Figure S31). Interestingly, the increase in the area of the Ni(II) to Ni(III) oxidation peak is paired with a shift to less anodic potentials, approaching the peak potential registered for Ni^0^ NCs (Figure S31). This entails that the formation of Ni(III) species on the surface of NiAs NCs-derived catalysts is less favored than on Ni^0^ NCs. A deeper discussion on the phenomenon is available in the Supporting Information. The decrease in the intrinsic activity of NiAs during operation and the shift in the Ni(II) to Ni(III) oxidation peak potential suggest that the NiAs surface is modified during operation, most likely leading to a Ni-rich surface.

**Figure 5:**
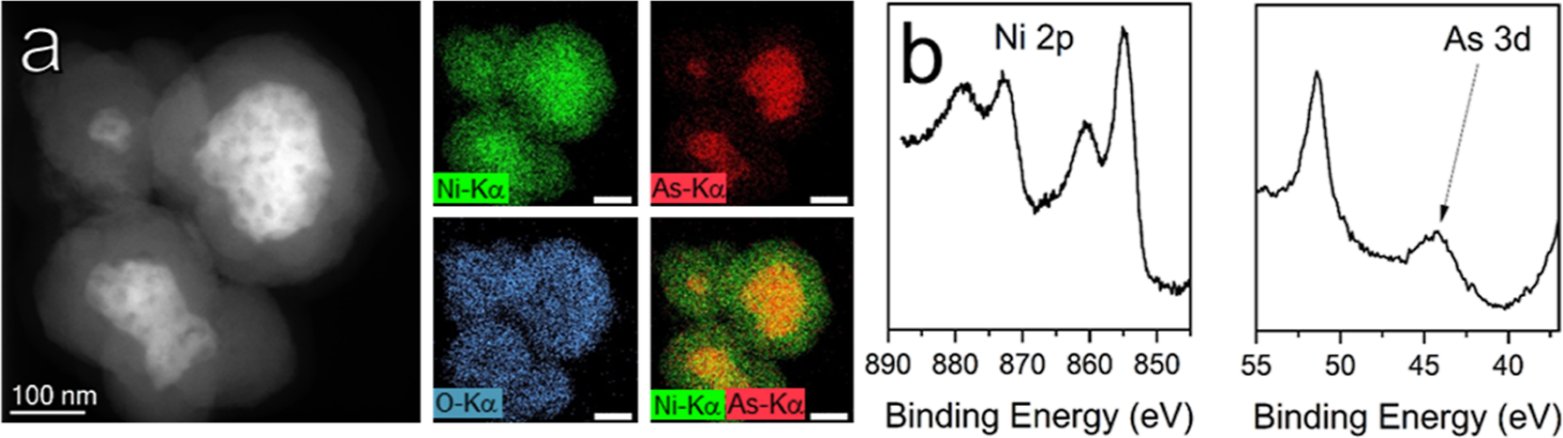
(a) HAADF-STEM image and related Ni, As, O, and Ni + As STEM–EDS elemental maps (scale bars in maps are 100 nm), (b) high-resolution XPS spectra of Ni 2p and As 3d regions collected on NiAs/Toray paper electrodes after 60 h of OER testing (chronopotentiometric scan, 10 mA cm_geo_^–2^).

To shed light on the catalyst modification during operation, electrodes were characterized after the chronoamperometric tests. The XRD pattern (Figure S35) of electrodes after operation still exhibits the typical reflections of the NiAs structure (again, along with graphite reflections stemming from the support), although the signal intensities in comparison with those of the support are considerably diminished, suggesting a decrease in the NiAs NCs loading upon reaction. However, XRD is a bulk technique and the residual presence of NiAs does not exclude the possibility of the formation of an amorphous shell of Ni oxy/hydroxides, as reported for Ni-metalloids alloys.^10^ SEM images of the used electrodes evidenced a pronounced agglomeration of the active phase, resulting in thread-like structures of NiAs, with size in the scale of micrometers (Figure S36). Correlated SEM–EDS spectra and maps (Figure S37) indicated an overall unbalance of the Ni to As atomic ratio (from the original 1:1 to a *ca.* 70:30 one, Table S10). Considering the retention of the NiAs phase observed by XRD, it is most likely that such a different atomic composition of aggregates stems from the formation of an As-depleted amorphous phase on the surface of NiAs NCs aggregates. Consistently, HAADF-STEM images, collected on NiAs NCs detached from the support by sonication, suggested the formation of core@shell structures, with a Ni-rich shell onto NiAs NCs aggregates (Figure 5a). STEM–EDS maps (Figures 5a and S38) are consistent with a Ni-rich shell composed of Ni oxy/hydroxides. The surface enrichment in Ni (i.e., surface depletion in As) is further confirmed by XPS (Figure 5b), from which a surface atomic ratio of Ni/As of *ca.* 92:8 is obtained. Moreover, XPS data show that Ni and As chemical environments at the surface of the active material were strongly modified upon operation. Indeed, the Ni 2p spectrum of the sample after OER shows signals consistent with the presence of Ni(OH)_2_,^36^ while the As 3d spectrum shows only a peak centered at *ca.* 44.1 eV, which might suggest the presence of As_2_O_3_.^37^ For both elements, the signals typical of NiAs completely disappeared, indicating that the NiAs phase is not present in the external 10 nm (i.e., probing depth of the technique) of the active material. The post-CP characterization is therefore consistent with the electrochemical data previously discussed. Ni(III) active sites in NiAs NCs exhibit an increased intrinsic activity toward OER with respect to those of Ni^0^ NCs (Figure 4b). This difference is most likely due to the electronic modulation operated by the presence of a NiAs core, highlighted by the Δ*E* in the Ni(II) to Ni(III) oxidation peaks in the two materials (Figure S31). The decrease in intrinsic activity (Figures 4b and S30) and the shift in the Ni(III) formation potential (Figure S31) observed upon operation are related to surface As depletion. Despite this phenomenon, the NiAs core of the catalyst is retained and imparts to the system an increased intrinsic OER activity in comparison with Ni^0^ NCs.

## Conclusions

In this study, we report the first colloidal synthesis of NiAs NCs featuring a platelet shape with a diameter of *ca.* 10 nm. The performances of NiAs NCs in typical electrochemical water splitting reactions, namely, acidic HER and alkaline OER, were studied in a three-electrode configuration cell. NiAs NCs exhibit the best intrinsic HER activity among TMAs reported to date despite presenting long-term stability issues and HER overpotentials and Tafel slopes larger than those of other PGM-free electrocatalysts (e.g., phosphides). NiAs NCs function as pre-catalysts for alkaline OER, with the actual active phase being represented by a shell of Ni oxy/hydroxides grown at the expenses of the starting NiAs NCs (with the formation of NiAs@Ni-oxy/hydroxides core@shell particles) under OER operative conditions. As a result, NiAs NCs featured an OER activity higher than that of benchmark Ni^0^ NCs. Noticeably, the OER performance, in terms of , is retained up to 60 h of continuous operation, with only a slight increase in the OER overpotential. In conclusion, NiAs NCs (and TMAs in general) hardly place themselves among PGM-free alternatives for acidic HER because of their modest activity and, especially, instability; on the other hand, NiAs NCs might represent an interesting matter of study in the field of OER pre-catalysts, following the pathway already outlined by other TM compounds (e.g., selenides).

## Supporting Information Available

Synthesis and characterization of NiAs NCs; characterization of pristine NiAs/Toray paper electrodes; electrochemical characterization methods; and acidic HER and alkaline OER electrocatalytic tests (PDF)
