# Supplementary material for: Colloidal Synthesis of Nickel Arsenide Nanocrystals for Electrochemical Water Splitting
Source: ACS Appl Energy Mater. 2022 Dec 23;6(1):151–9. doi: 10.1021/acsaem.2c02698 (PMC9832430; doi:10.1021/acsaem.2c02698)
Supplement: Supplementary file 1 — ae2c02698_si_001.pdf [file ae2c02698_si_001.pdf]

Supporting Information to:

Colloidal Synthesis of Nickel Arsenide Nanocrystals for Electrochemical Water Splitting

Fulvio Bellato[a,b], Michele Ferri[a], Abinaya Annamalai[a,b], Mirko Prato[a], Luca Leoncino[a], Rosaria Brescia[a], Luca de Trizio[a], Liberato Manna[a,]*

[a] Istituto Italiano di Tecnologia (IIT), Via Morego 30, Genova, 16163, Italy

[b] Università degli studi di Genova (UniGe), Via Dodecaneso 31, Genova, 16146, Italy

Corresponding authors: Prof. Liberato Manna – liberato.manna@iit.it

Dr. Michele Ferri – michele.ferri@iit.it

Content of Supplementary Information

1.1 Synthesis and characterization of NiAs NCs

1.2 Characterization of pristine NiAs/Toray paper electrodes

1.3 Electrochemical characterization

1.4 Acidic HER: supplementary electrochemical data

    1.4a Alkaline HER: additional performance study

1.5 Alkaline OER: supplementary electrochemical data

1.1 Synthesis and characterization of NiAs NCs

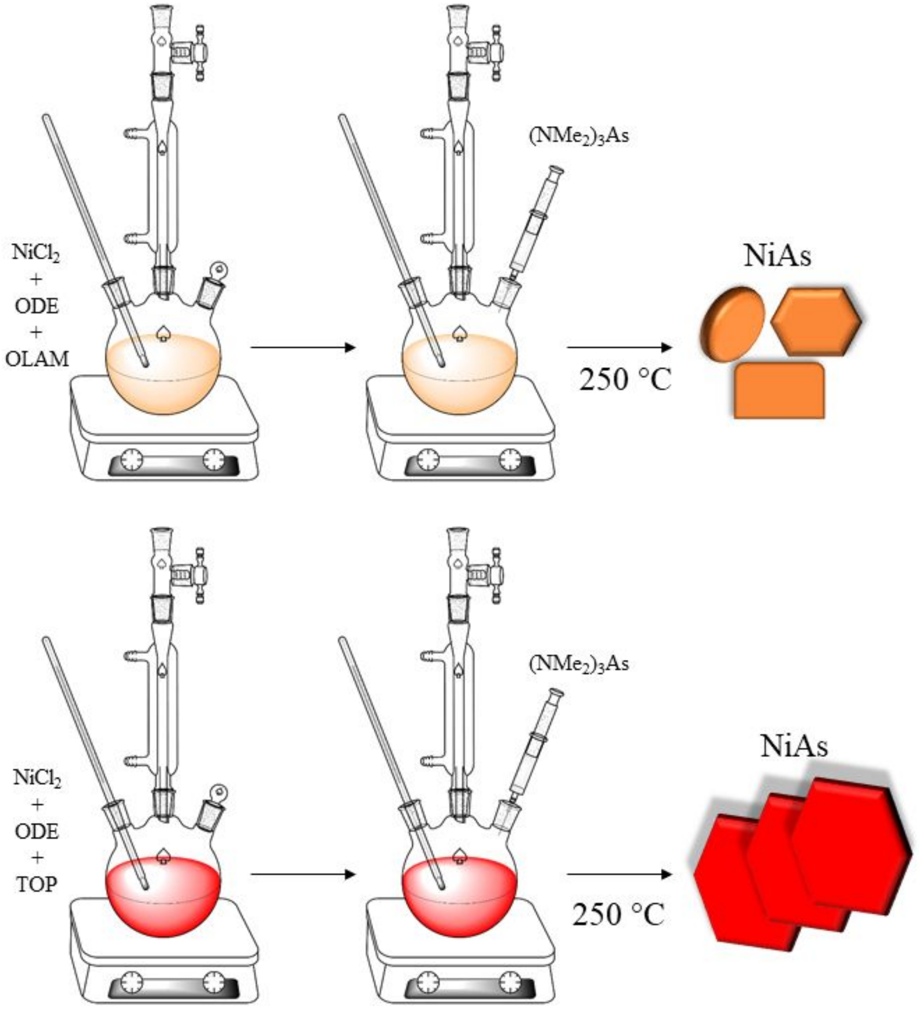

Figure S1: Graphical depiction of the syntheses of NiAs NCs with different ligands, namely OLAM (top) and TOP (bottom)

Table S1: List of the NiAs NCs synthetic attempts, gathering all the Ni precursors, ligands/solvents and operative conditions explored in the study. The optimized reaction conditions for OLAM- and TOP-NiAs NCs are written in bold. Acronyms are defined in the following paragraph. All syntheses utilized tris(dimethylamino)arsine as As precursor.

Ni precursors	Solvents	Reaction Temperature (°C)	Time reaction (min)
**$NiCl_2$**	ODE OA	190	5
		220	
	**ODE OLAM**	220	1
			5
			10
		**250**	**1**[a]
			3
			5
			10
	OLAM	220	1
		250	1
			3
			5
			10
		280	1
	OCTAM	250	1
			15
			30
	TOPO	250	1
			15
			30
	TOP	250	1
	**ODE TOP**	**250**	**1**
$Ni(acac)_2$	ODE TOP	250	1
	ODE OLAM	220	5
		250	1
			15
			30

[a] *Reaction also scaled-up by heat-up method*

Additional comments to the NiAs NCs synthesis optimization

Throughout this study, several ligands such as oleic acid (OA), oleylamine (OLAM), octadecylamine (OCTAM), trioctylphosphine oxide (TOPO) and trioctylphosphine (TOP) have been tested in order to optimize the NiAs synthesis route. In the following, the outcomes of all synthetic approaches are commented in the same order in which experiments are presented in Table S1.

At first, $NiCl_2$ and tris(dimethylamino)arsine have been chosen as Ni and As precursors, respectively.

OA in ODE: The interaction between the carboxylic group of OA and the aminoarsine leads the formation of As_2O_3 (Figure S2a). A possible reason for the formation of As_2O_3 might be water formation in the reaction flask (through the condensation reaction between OA and aminoarsine) that, combined with the high temperature, leads to the decomposition of the As precursor. To avoid the formation of water as by-product of the reaction, OLAM might be used as ligand.

OLAM in ODE: Performing the reaction at 220˚C for 1 minute yields a NiAs bi-phasic sample composed by nickeline (NiAs) and maucherite ($Ni_{11}As_8$) (Figure S2b). The maucherite phase completely converts into nickeline at longer reaction times (i.e. 10 minutes). By raising the temperature to 250˚C we obtain NiAs pure phase samples regardless the reaction time (Figure S2c, Figure S3e,f). Also, OLAM is a ligand that may be easily handled outside the glovebox, therefore up-scaling of the reaction by the heat-up method is possible under these reaction conditions. This leads to the possibility to synthesize large quantities of NiAs NCs per batch. However, large (34.0 ± 0.5 nm, inset in Figure S3e) and irregular NCs are obtained through this synthetic approach. Note that the synthetic procedure followed when using OLAM as capping agent is identical to the one reported in the Experimental Section but replacing TOP with the same volume of OLAM (70% technical grade, purchased from Sigma-Aldrich).

OLAM: The possibility to use OLAM as both solvent and ligand has also been explored, performing the synthesis at different reaction times (Figure S2d) or temperatures (Figure S2e). Regardless the reaction time and/or temperature set, all samples exhibited multiple NiAs phases.

OCTAM and TOPO: When OCTAM (Figure S2f) or TOPO (Figure S2g) are used as solvent/ligand, the patterns obtained are not clear and therefore suggest poor phase purity.

TOP and TOP in ODE: The use of TOP as both ligand and solvent (250˚C, 1 minute) result in the formation of NiAs but with a non-negligible contamination by As_2O_3 (Figure S2h). On the other hand, the use of TOP in ODE (250˚C, 1 minute) allows to obtain pure phase (Figure S2i), *ca.* 10 nm NiAs nanodisks.

When switching to a different Ni precursor (i.e. $Ni(acac)_2$), the synthesis is unsuccessful irrespective of the solvents, ligands, reaction temperatures and/or times explored (Figures S2j,k,l).

Sample TEM images of the products of unsuccessful synthetic protocols are reported in Figure S3a-d. TEM images of pure phase but irregular shaped NiAs NCs, obtained using OLAM in ODE, are also reported (Figure S3e,f).

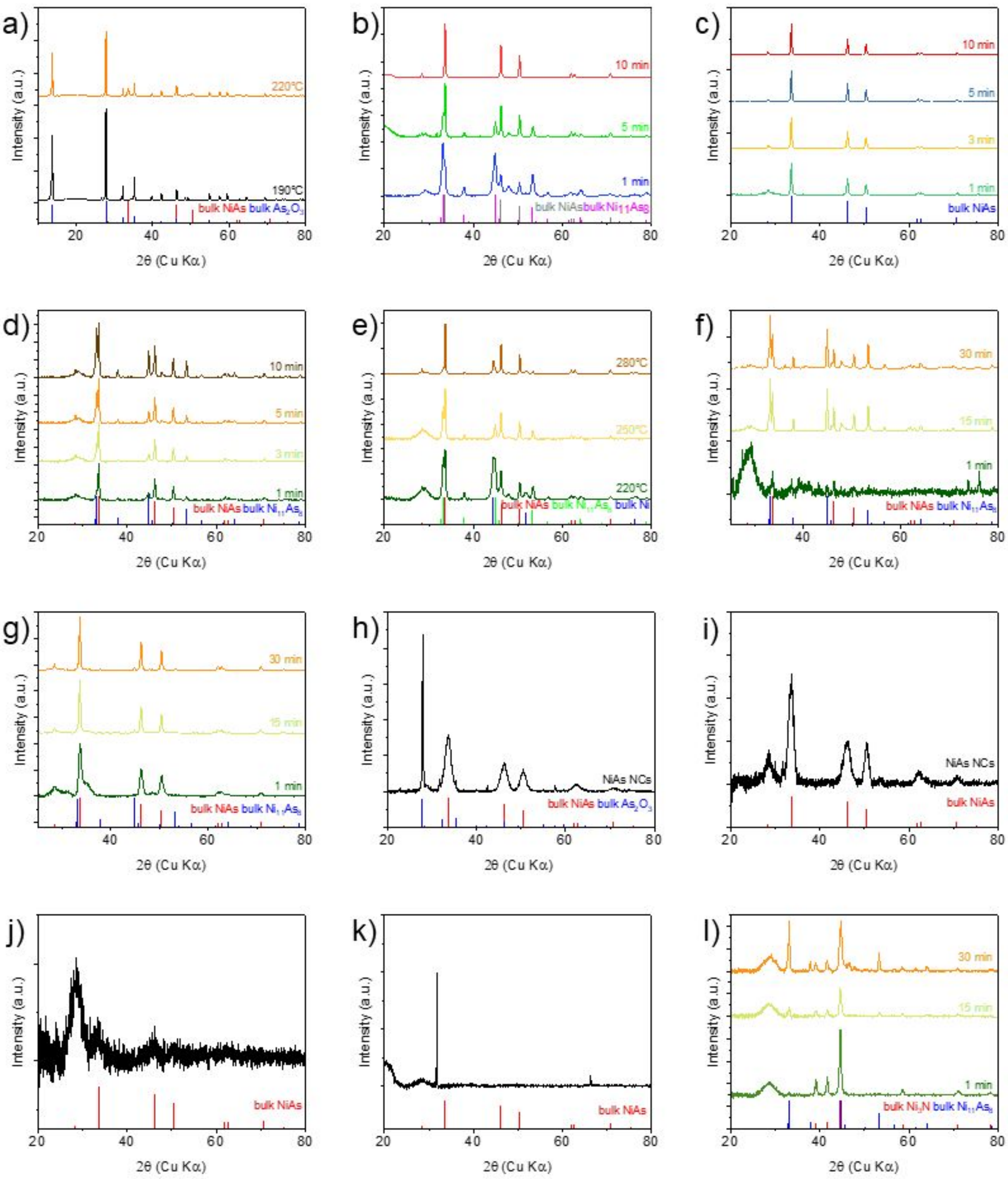

Figure S2: XRD patterns of the samples obtained from the synthesis protocol reported in Table S1. Synthetic attempts performed using $NiCl_2$ as precursor: a) OA in ODE, 5 minutes; b) OLAM in ODE, 220°C; c) OLAM in ODE, 250°C; d) OLAM, 220°C; e) OLAM, 1 minute; f) OCTAM, 250°C; g) TOPO, 250°C; h) TOP, 250°C, 1 minute; i) TOP in ODE, 250°C, 1 minute. Synthetic attempts performed using $Ni(acac)_2$ as precursor: j) TOP in ODE; k) OLAM in ODE, 220°C; l) OLAM in ODE, 250°C.

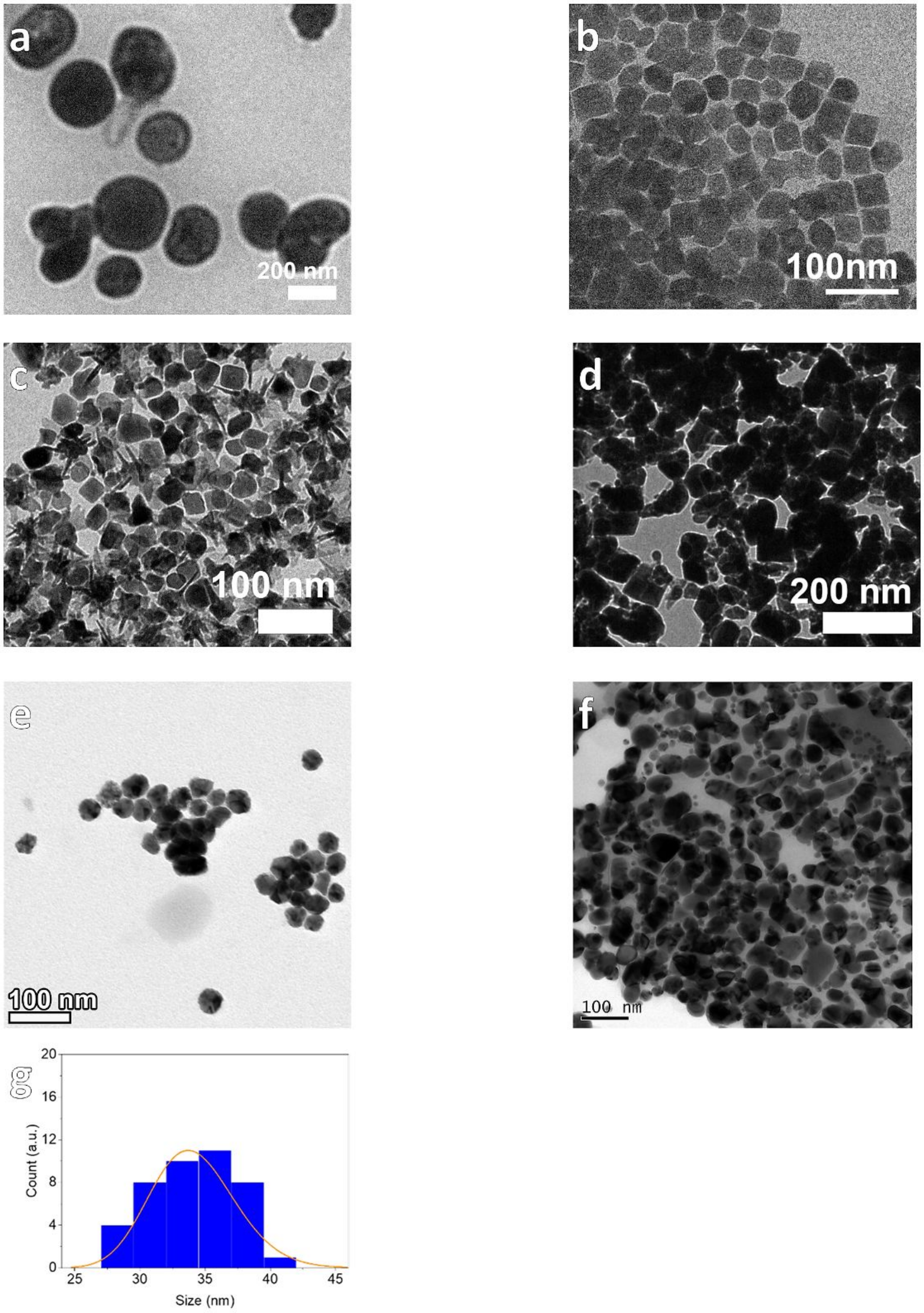

Figure S3: Sample TEM images of the products of unsuccessful synthetic protocols: a) OA in ODE; b) OLAM in ODE, 220°C; c) OLAM; d) TOP; e) OLAM in ODE, 250°C, 1 minute, f) OLAM in ODE, 250°C, 10 minutes; g) particles size distribution related to (e).

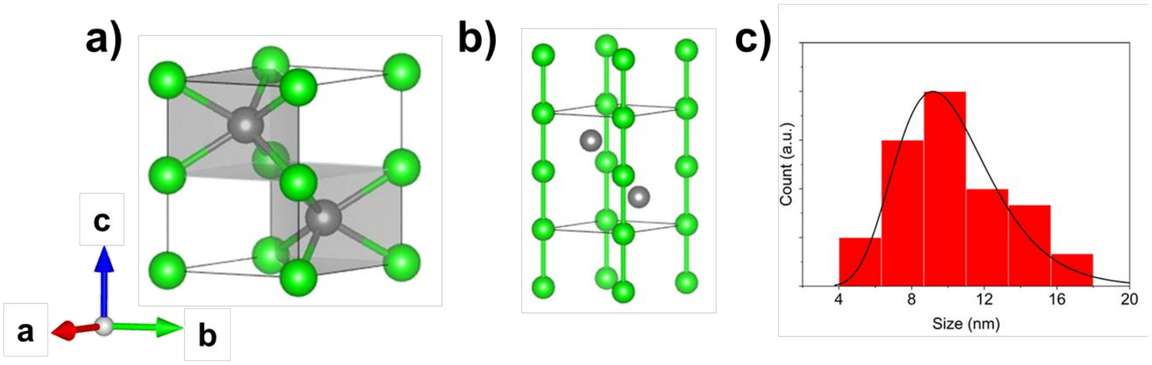

Figure S4: a) Representation of the prismatic triangular coordination of As (gray) anions with Ni (green) cations in NiAs; b) Ni-Ni bonds along the c (001) direction in the NiAs structure. The color code is the same used in (a); c) Size distribution of TOP-synthesized NiAs NCs (number of NCs measured ≈ 150).

Table S2: Indexed NiAs reflections

**Crystal plane (*hkl*)**	**° (2θ)**
1,0,1	33.650°
1,0,2	46.201
1,1,0	50.386

Table S3: Concentration (ppm and ppmmol) and molar ratio of Ni and As as obtained from ICP-OES analyses. Each value reported is the averaged one obtained from three different analyses

	Analyte concentration				**Molar ratio**
	ppm		*mmol L^{-1}*		
	**Ni**	**As**	**Ni**	**As**	**Ni/As**
**TOP-NiAs NCs**	0,051	0,070	8,61E-4	9,30E-4	0,93
**OLAM-NiAs NCs**	0,222	0,283	3,78E-3	3,77E-3	1,00

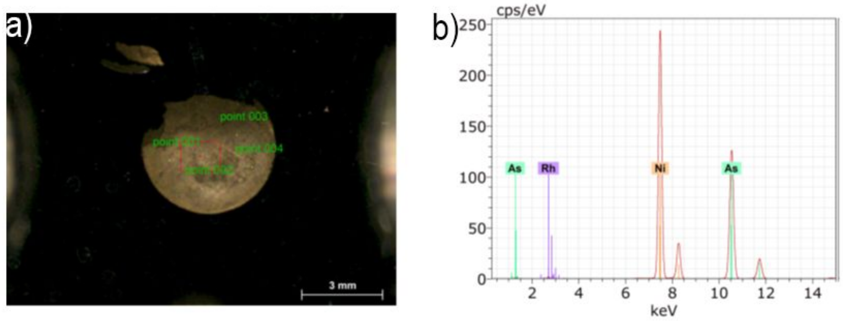

c)

Point	Atomic % by XRF	
	Ni	As
1	48,70	51,30
2	48,99	51,01
3	48,81	51,19
4	48,76	51,24

Figure S5: (a) Drop-cast NiAs NCs suspension on XRF substrate, (b) related spectrum and (c) atomic Ni to As ratio as obtained from XRF on the spots indicated in (a).

1.2 Characterization of pristine NiAs/Toray paper electrodes

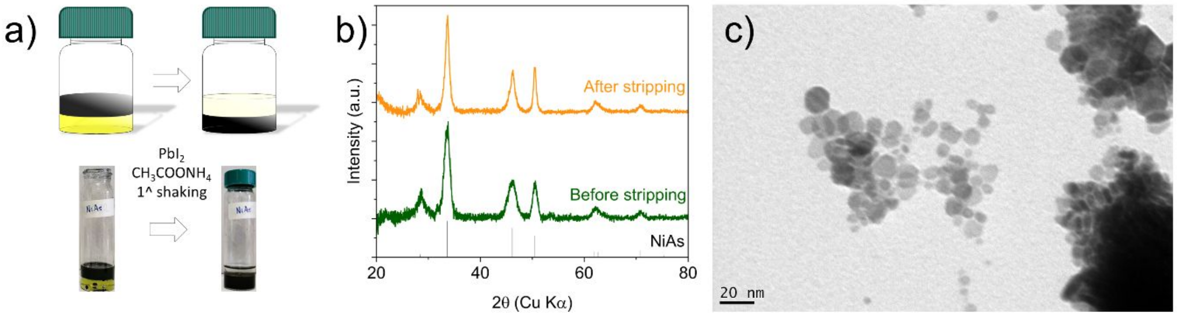

Figure S6: (a) Graphical depiction of the stripping process, (b) XRD patterns of the NiAs NCs before and after ligand stripping and (c) TEM image of ligand-stripped NiAs NCs.

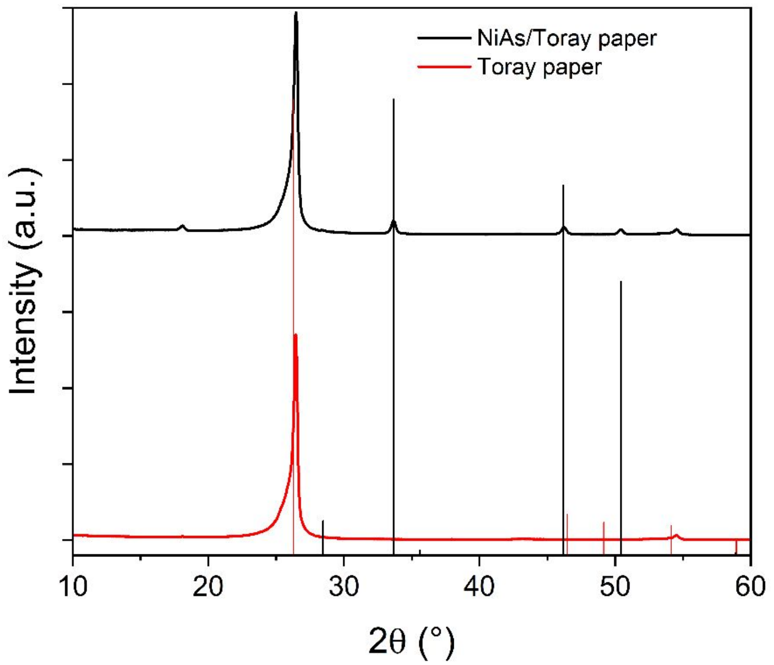

Figure S7: XRD patterns of supported NiAs NCs (black line) and pristine Toray paper (red line). Black and red sticks represent reference reflections of NiAs (hexagonal, ICSD 611040) and graphite (ICSD 76767), respectively.

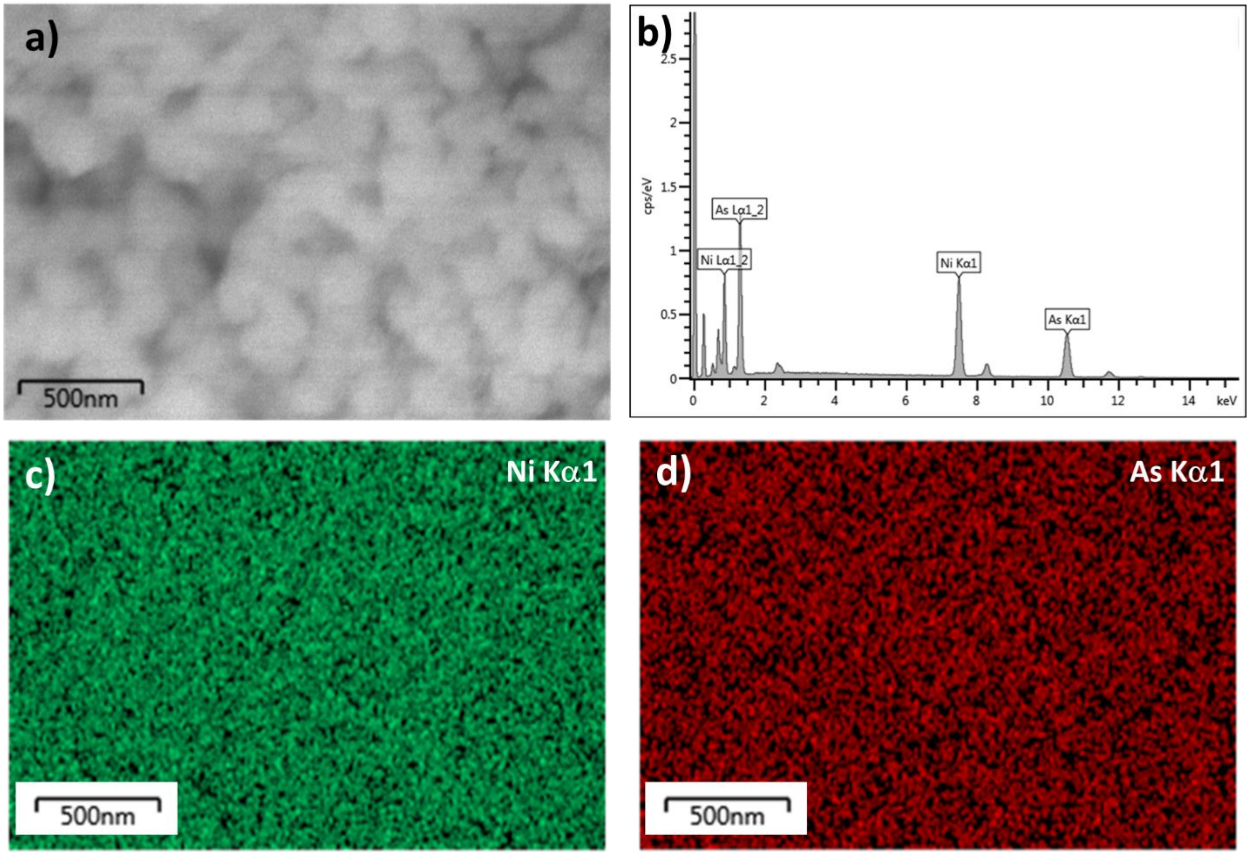

Figure S8: (a) FE-SEM images, (b) SEM-EDS spectrum and (c, d) SEM-EDS maps of Ni and As collected on pristine NiAs/Toray paper electrodes.

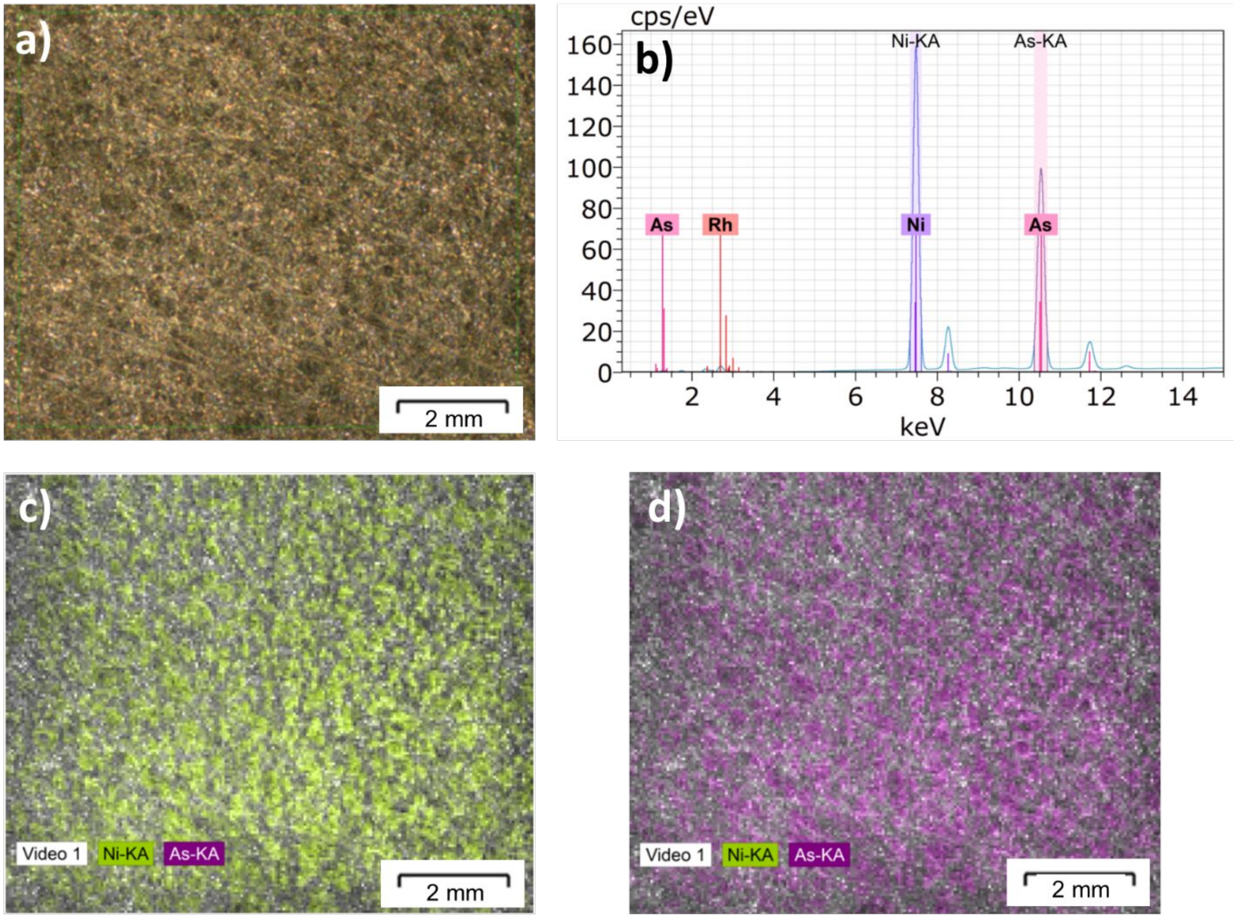

Figure S9: (a) Picture of analyzed area and related (b) XRF spectrum and (c, d) XRF maps of Ni and As collected on pristine NiAs/Toray paper electrodes.

Table S4: Average atomic ratio of Ni and As as detected by XRF on pristine NiAs/Toray paper electrodes

	**Atomic %**
**Ni-Kα**	49.67
**As-Kα**	50.33
**Total**	100

1.3 Electrochemical characterization

The following table reports the testing routines applied for HER and OER, providing all the significant analytical parameters. Potential ranges are reported versus the reference electrode (3 M KCl Ag/AgCl). Regarding the determination of ECSA by double-layer capacitance method and the use of potentiostatic techniques to assess Tafel slopes, additional information may be found in the dedicated paragraphs. However, since the accurate determination of both ECSA and Tafel slope require ad-hoc optimization of the electrochemical, the reader is recommended to refer to the articles indicated in the following table.

Table S5: Electrochemical routines followed for assessing HER (acidic) and OER (alkaline) catalytic performance of NiAs

	Electrochemical technique	HER	OER
1	CV – Electrode conditioning	E range: 0 to -1.2 V vs Ag/AgCl<br>E step: 5 mV<br>20 cycles<br>Scan rate: 100 mV s^{-1}	E range: 0 to 1.2 V vs Ag/AgCl<br>E step: 1 mV<br>50 cycles<br>Scan rate: 100 mV s^{-1}
2	PEIS	E: 0.7 V vs Ag/AgCl<br>Freq. range: 50000 to 0.1 Hz<br>ΔE: 0.1 V	E: 0.3 V vs Ag/AgCl<br>Freq. range: 100000 to 0.1 Hz<br>ΔE: 0.05 V
3	CV - ECSA determination	Refer to ([1]) and Figure S13<br>Flat standard: CoP_3<br>C_{CoP3}: 60 μF cm^{-2} per cm^2_{ECSA}	-
4	LSV	E range: -0.2 to -1.2 V vs Ag/AgCl	E range: 0.3 to 1.0 V vs Ag/AgCl

		E step: 1 mV<br><br>Scan rate: 2 mV s^{-1}	E step: 1 mV<br><br>Scan rate: 2 mV s^{-1}
5	CA – Tafel slope determination	Refer to ([2]) and Figure S14	Refer to ([2]) and Figure S33
6	CP – ± 10 mA cm^{-2}_{Geo}	I: -2.5 mA (-10 mA cm^{-2}_{Geo})	I: 2.5 mA (10 mA cm^{-2}_{Geo})
7	LSV	As reported in point 4	As reported in point 4
8	PEIS	As reported in point 2	As reported in point 2

ECSA determination by C_{dl} method

To compare the intrinsic electrochemical activity of different systems, the electrochemically active surface area of the electrodes may be estimated through the measure of its specific capacitance. Briefly, CVs of the studied electrode are collected in a non-faradaic region by applying different potential scan rates. The resulting capacitive current is taken as the mean (absolute value) of cathodic and anodic currents registered in the middle of the potential window. Such averaged capacitive current is then plotted against the applied potential scanrate and specific capacitance regressed. Conversion of the specific capacitance of the studied electrode into ECSA is generally performed using the specific capacitance for a flat standard with a 1 cm^2.

$$ECSA = \frac{Electrode\ specific\ capacitance\ (\mu F\ cm^{-2}),\ measured\ by\ C_{dl}\ method}{Flat\ standard\ specific\ capacitance\ (\mu F\ cm^{-2}\ per\ cm^2_{ECSA})}$$

In the present study, in accordance with Gauthier et al.[3], a thin film of CoP_3, exhibiting a capacitance of 60 μF cm^{-2} per cm^2_{ECSA} has been taken as standard. As previously discussed, ECSA determination by the C_{dl} method endows several steps, each one of them to be optimized to the system under study to achieve reliable data. The authors recommend to the reader to refer to dedicated papers.[1]

Tafel slope regression from potentio/galvanostatic methods

As discussed in the main text, the regression of Tafel slopes from data collected by means of potentiodynamic techniques is hindered by the unavoidable contribution of capacitive currents. With the aim to minimize such currents, a rapid and effective method for assessing Tafel slopes consist in collecting i vs E data by means of potentio/galvanostatic methods [2], i.e. chronoamperometric or chronopotentiometric techinques.

Briefly, when using a chronoamperometric technique, several fixed potential steps are set, resulting in a decreasing staircase-like trend of the registered current. The initial step is at the highest potential applied as to promote the formation of the electric double layer at the electrode-electrolyte interface. The acquistion time for each step is set as to achieve a stationary current, therefore eliminating any capacitive contribution. The same procedure may be applied when using a chronopotentiometric technique, in this case starting from higher current densities, resulting in a staircase-like potential trend. Data for Tafel slope regression are collected by averaging the last few points registered at the end of each step (where capacitive currents are negligible) and then plot as overpotentials versus log i, obtaining the typical Tafel plots. Since each electrochemical system possesses unique capacitive and/or inductive behavior, in addition to peculiar catalytic activity, ad-hoc optimization of the operative parameters (especially the timescale of potentio/galvanostatic steps) has be to carried out.[2] Insets in Figures S14b and S32b report the traces of chronoamperometric/chronopotentiometric scans from which the data for Tafel slope regression has been extracted.

1.4 Acidic HER: electrochemical data

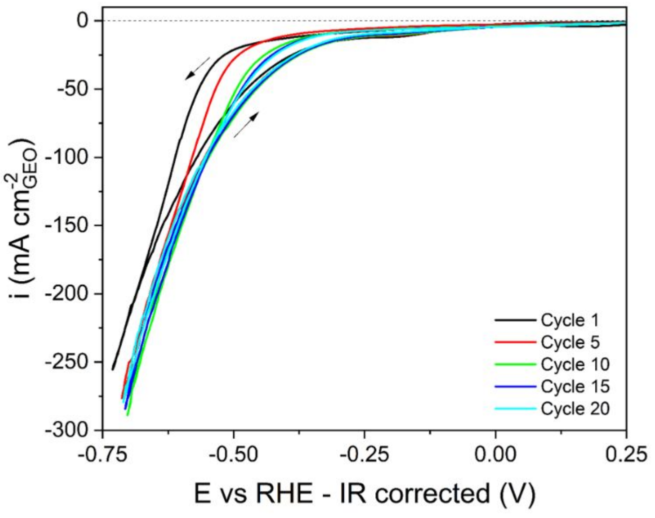

Figure S10: Sample conditioning cyclovoltammetric curves (scan rate = 100 mV s^{-1}) of NiAs/Toray paper electrodes

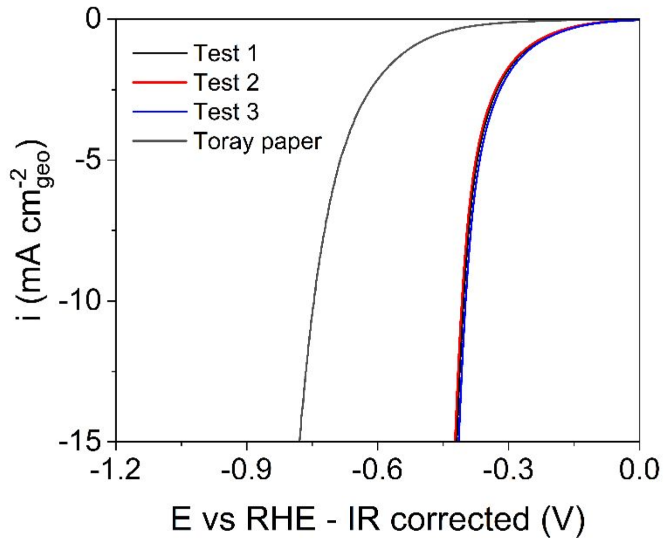

Figure S11: Replicated LSV curves (scan rate = 2 mV s^{-1}) registered on three independent NiAs/Toray paper electrodes in comparison with the bare support.

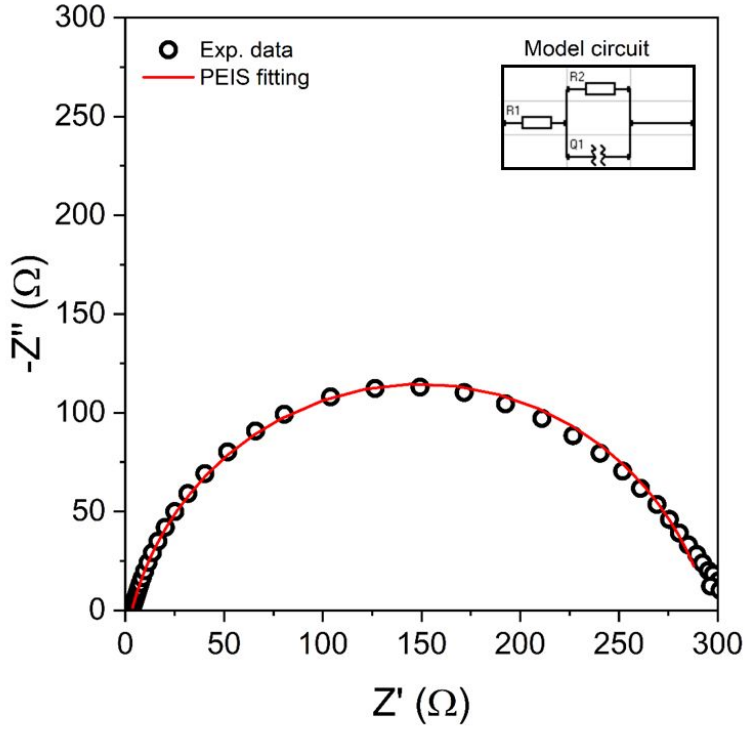

Figure S12: Sample Nyquist plot obtained by PEIS on the NiAs/Toray paper electrode. In the inset, the model circuit used for modeling experimental data and evaluate the system uncompensated resistance R_u

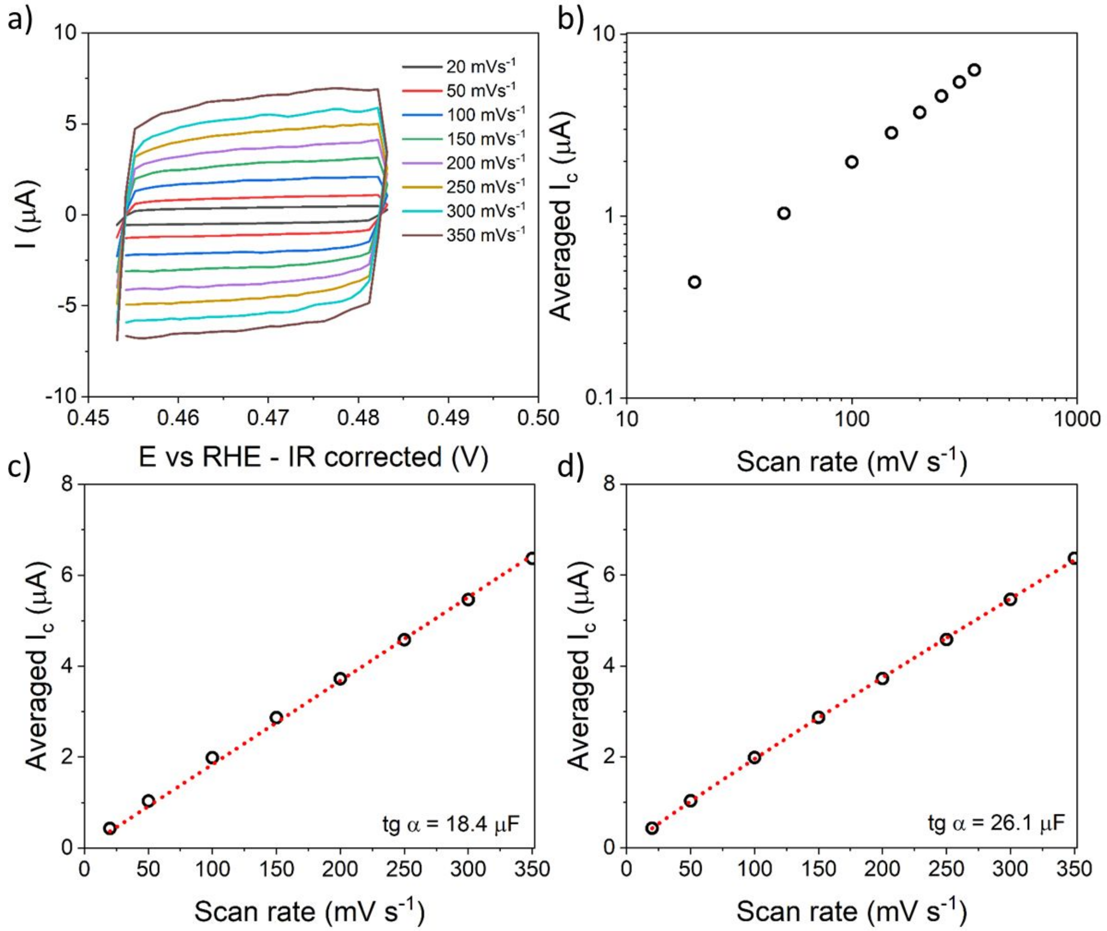

Figure S13: (a) Cyclovoltammetric curves collected on NiAs/Toray paper electrodes in non-faradaic region at different scan rates; (b) Logarithmic plot of the average I_C, determined from CVs reported in (a) at 470 mV vs RHE (IR-corrected) versus the applied voltage scan rate; (c) linear and (d) allometric regression of the averaged I_C versus the applied voltage scan rate, yielding the specific capacitance of NiAs/Toray paper electrodes.

Table S6: Model equations, regressed parameters and adjusted R^2 coefficient obtained from linear and allometric regression of data reported in Figure S13c and d.

	**Regression model**	
	Linear	Allometric
**Equation**	y = a + bx	$y = bx^n$
**Intercept (a parameter)**	-	-
**Slope (b parameter, [μF])**	0.0184 ± 0.0002	0.0261 ± 0.0010
**Adjusted R^2**	0.9994	0.9998

Further discussion on ECSA determination by C_{dl} method

Normalization of the delivered current by the electrochemically active surface area (ECSA) allows a fair comparison of the electrocatalytic activity of different materials; however, accurately determining ECSA might be challenging, as proven by the numerous articles and reviews on the topic.[4,5] Among all methods proposed for ECSA determination, the double-layer capacitance is the most used one, mainly because of its universality and simplicity.[1] On the other hand, the accuracy and reproducibility of this method is very poor when compared to other methods (e.g. underpotential deposition of metals and probe molecules stripping methods).[4] The rather limited literature on TMAs generally reports ECSA-corrected data by applying the double-layer capacitance method,[3] so we followed the same approach for our samples.

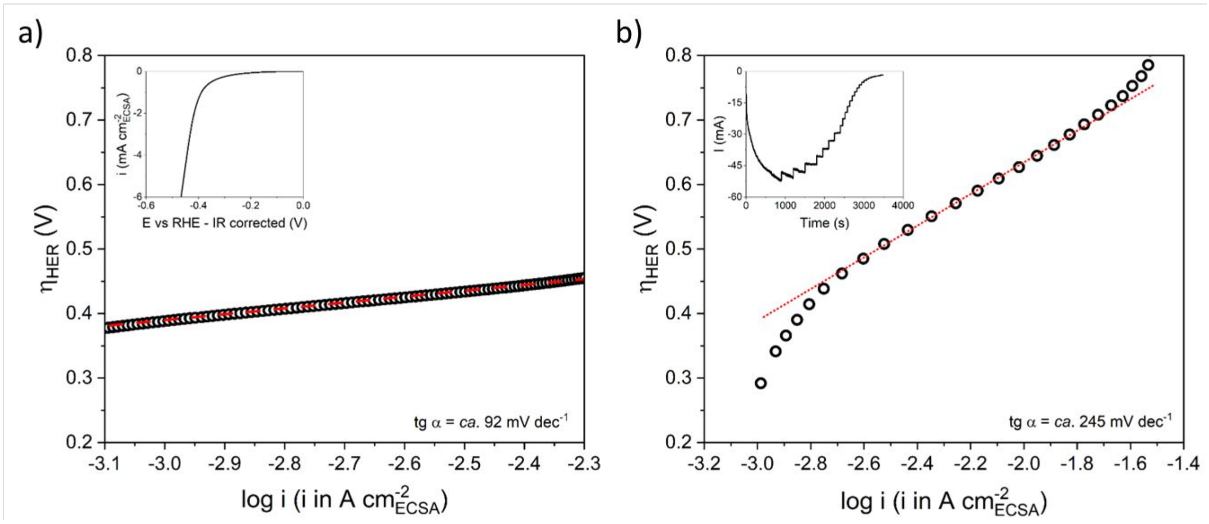

Figure S14: Tafel slopes extracted from (a) linear sweep voltammetry and (b) chronopotentiometric tests. Electrochemical traces from which overpotential versus log i data have been obtained are reported in the insets

Extended discussion on HER Tafel slopes determination and significance

From a kinetic and mechanistic point of view, the measurement of Tafel slopes describes the current-voltage relationship for a specific catalyst and allows assessing the rate-determining step of HER on the latter [6]. NiAs Tafel slope, obtained from LSVs, stands around 92 mV dec^{-1} (Figure S14a), in line with those reported for MoAs and CoAs (*ca.* 75 mV dec^{-1}). These results indicate the Volmer step (Scheme 1, theoretical Tafel slope *ca.* 120 mV dec^{-1}) as the most likely rate-determining step of HER on TMAs (Heyrovsky and Tafel steps exhibit theoretical Tafel slopes around 40 and 30 mV dec^{-1}, respectively [6]). Nevertheless, it must be stated that the use of potentiodynamic techniques is not recommended for the determination of Tafel slopes, as the inevitable contribution of capacitive currents results in unreliable data [2]. When regressing the Tafel slopes from a potentiostatic approach (Figure S14b), NiAs exhibits values higher than 120 mV dec^{-1} (*ca.* 245 mV dec^{-1}). When deriving Tafel slopes from first principles [7], it appears evident that values > 120 mV dec^{-1} do not possess any physical meaning, at least when considering the most straightforward reaction mechanism depicted in Scheme 1. Nonetheless, several authors reported HER Tafel slopes > 120 mV dec-

[1] [8,9], claiming that those values stem from charge transfer coefficients (α) being lower than the postulated ones (α = 0.5 for Volmer and Heyrovsky steps and α = 0 for Tafel step). It should be also stressed that atypical α values may arise from the complex nature of reaction mechanisms, as commonly reported for electrocatalytic oxidation of organic molecules.[10] Taking into account all these considerations and acknowledging that the theoretical underpinnings of Tafel analysis do not hold universally,[2,6] we may only state that the most reliable 245 mV dec^{-1} slope determined for NiAs accounts for a hindered HER process on this catalyst and (most likely) on TMAs in general. It must also be stressed out that the measurement of Tafel slopes might be heavily affected by the intrinsic instability of NiAs NCs under acidic HER conditions, as discussed in the main text.

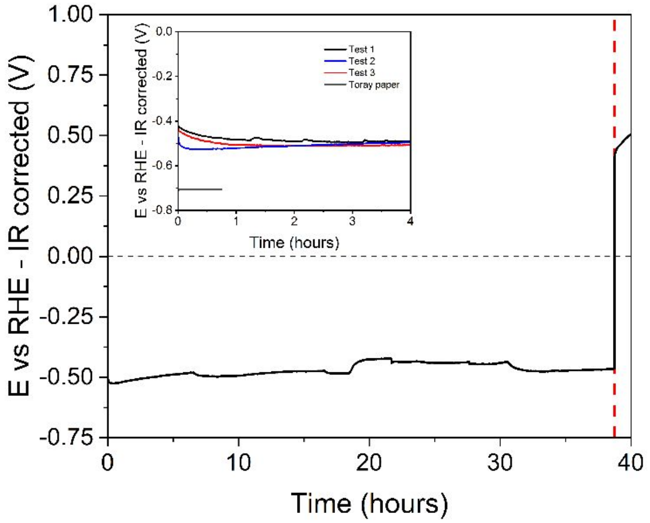

Figure S15: 40-hours long chronopotentiometric measurement at -10 mA cm_{geo}^{-2}. The vertical red dashed line indicates the time of electrode failure (measure overloading). In the inset, repetitive 4 hours-long CP tests.

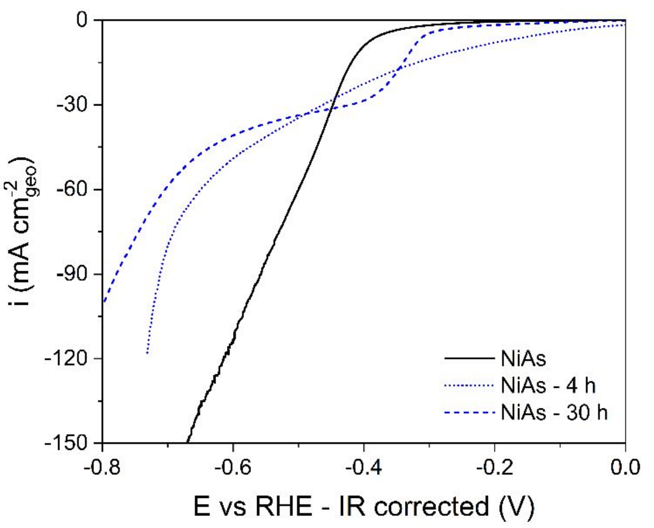

Figure S16: Linear sweep voltammetries (scan rate = 2 mV s^{-1}) collected on pristine (solid line) and used (dashed line) NiAs/Toray paper electrodes

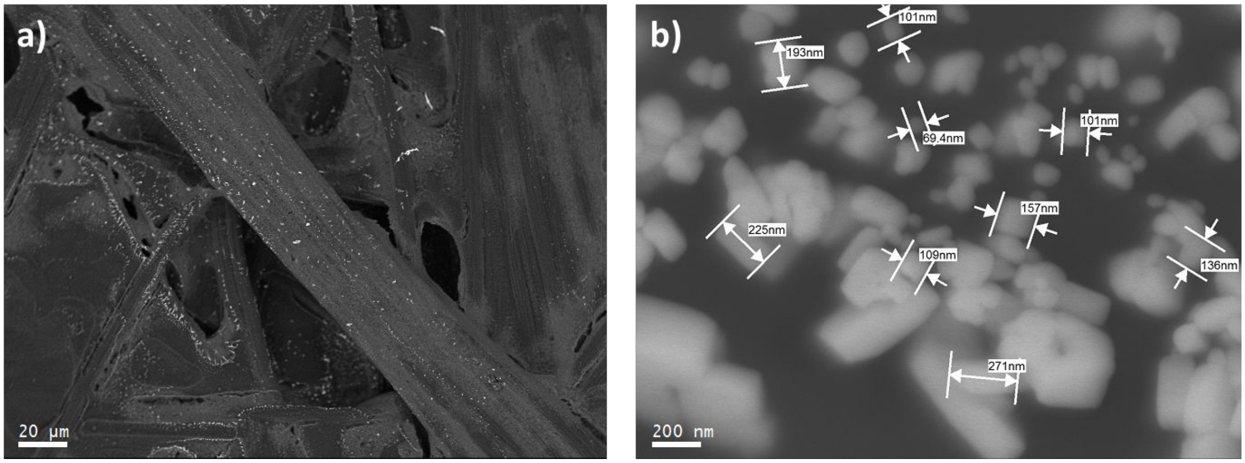

Figure S17: (a, b) FE-SEM micrographs of NiAs/Toray paper electrodes after a 40 hours-long HER chronopotentiometric test (-10 mA cm_{geo}^{-2}).

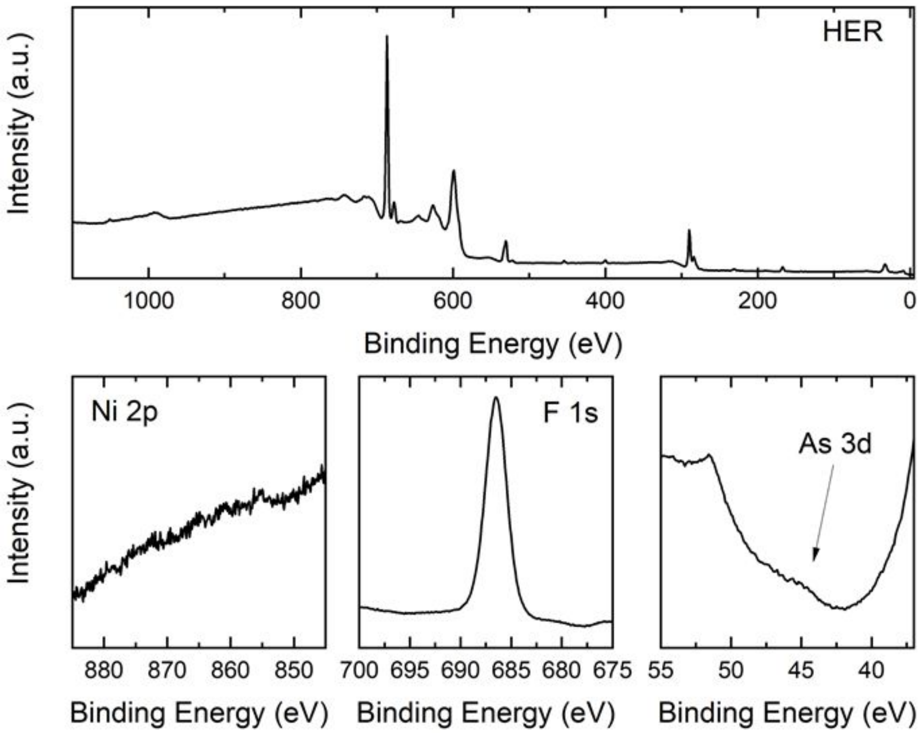

Figure S18: XPS survey (top) and high resolution XPS spectra of Ni 2p, F 1s and As 3d regions (bottom) collected on NiAs/Toray paper electrodes after 40-hours long chronopotentiometric scans. Atomic surface concentrations of Ni, As and F are 0.08, 0.01 and 99.91 at.% respectively, with F contribution being due to Nafion.

1.4a Alkaline HER: additional performance study

In the following, the NiAs performance under HER alkaline conditions is reported. The whole study has been carried out according to the standard electrochemical procedures reported previously (see the Experimental section in the main text and Table S5). The only differences from acidic HER testing are related to the electrolyte nature (being in this case 1 M aqueous KOH, treated with Chelex resins) and the potential window under study (when the latter is considered versus the Ag/AgCl reference electrode as it is in Table S5). However, all voltages are reported vs RHE, allowing the reader to retrieve the applied potentials vs Ag/AgCl (see the Experimental section in the main text).

Brief comments after each figure/table will summarize the main outcomes of this investigation.

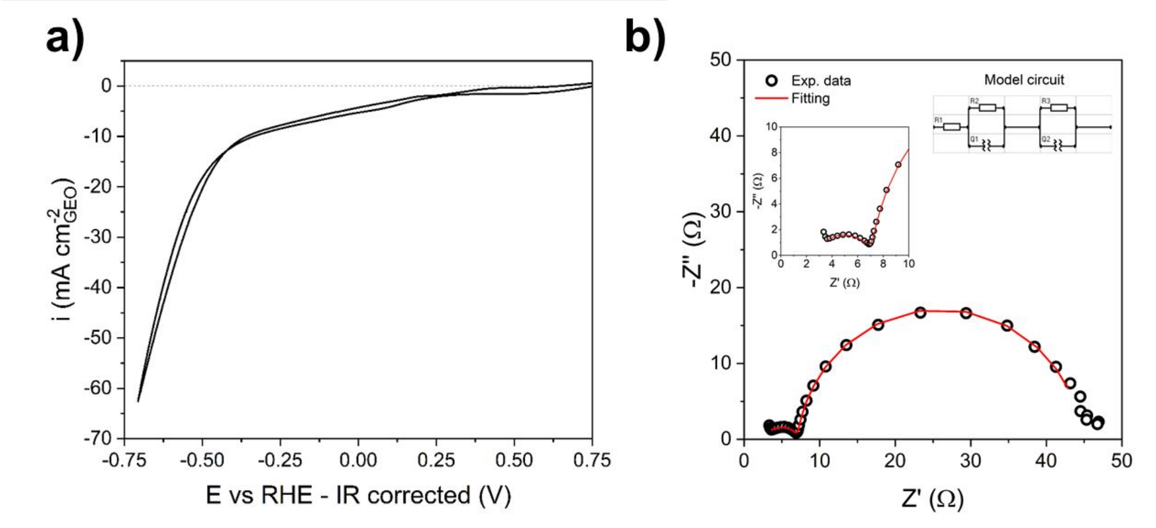

Figure S19: a) Sample cyclovoltammetric curve (scan rate = 100 mV s^{-1}) and b) sample PEIS, collected in the faradaic region (E = -0.5 V vs RHE), obtained on NiAs/Toray paper electrodes under alkaline conditions (1 M KOH)

The CVs collected on NiAs/Toray paper electrodes under alkaline conditions reveal the clear presence of two distinct cathodic phenomena, resulting in a double slope of the cathodic branch of the voltammogram

(Figure S19a). The first phenomenon, taking place for applied potentials lower than *ca.* 0.25 V vs RHE, might be represented by the reduction of surface carboxyl and hydroxyl groups of the carbon-based support. On the other hand, the HER onset corresponds to the inflection detected at ca. -0.38 V vs RHE. PEIS, collected in the faradaic region, consistently displays a double semicircle in the Nyquist plot (Figure S19b), indicative of two simultaneous faradaic reactions (precisely support reduction and HER on NiAs).

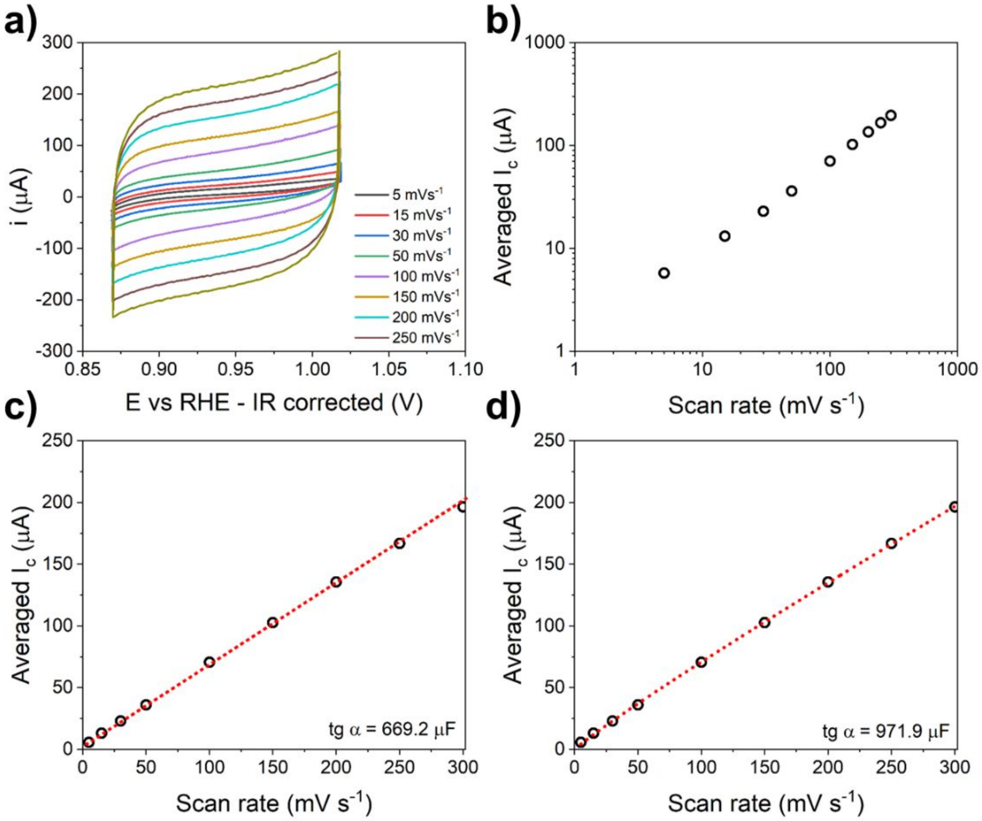

Figure S20: (a) Cyclovoltammetric curves collected on NiAs/Toray paper electrodes in non-faradaic region at different scan rates; (b) Logarithmic plot of the average I_C, determined from CVs reported in (a) at 0.95 V vs RHE (IR-corrected) versus the applied voltage scan rate; (c) linear and (d) allometric regression of the averaged I_C versus the applied voltage scan rate, yielding the specific capacitance of NiAs/Toray paper electrodes.

Table S7: Model equations, regressed parameters and adjusted R^2 coefficient obtained from linear and allometric regression of data reported in Figure S20c and d

	**Regression model**	
	Linear	Allometric
**Equation**	y = a + bx	$y = bx^n$
**Intercept (a parameter)**	-	-
**Slope (b parameter, [μF])**	0.66923 ± 0.00633	0.97192 ± 0.0370
**Adjusted R^2**	0.9992	0.9998

Figure S20 and Table S7 report the details of the ECSA estimation of NiAs/Toray paper electrodes under alkaline conditions. The same assumptions and limitation, discussed above for the ECSA determination by the C_{dl} method under acidic conditions, hold. It is interesting to notice how the double-layer capacitances measured on the same electrodes under alkaline environment are markedly larger than those recorded under acidic conditions (*ca.* 1 mF vs *ca.* 26 μF, according to allometric regression). As electrodes have been crafted following the same procedure, this result highlights how stable NiAs is under alkaline conditions in comparison with what observed at acidic pH (i.e. NiAs dissolution, leading to a drop in the measured C_{dl}).

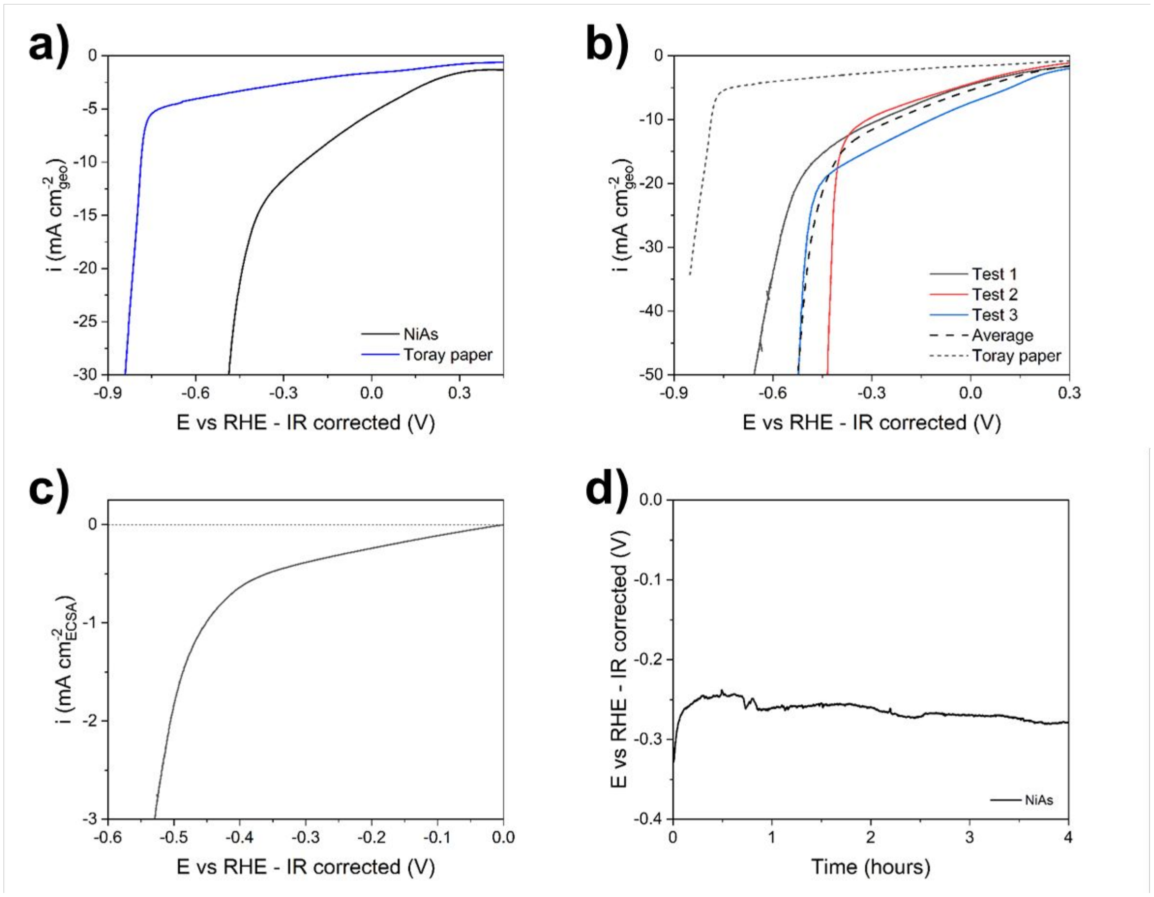

Figure S21: Evaluation of the electrochemical HER activity of NiAs NCs under alkaline conditions. Linear sweep voltammetries (scan rate = 2 mV s^{-1}) of NiAs and the bare support, displayed as potential vs (a) geometrical and (c) ECSA-corrected current densities. (b) LSV curves collected on NiAs/Toray paper electrodes and related average curve. (d) 4-hours long chronopotentiometric measurement (average) at -10 mA cm_{geo}^{-2}

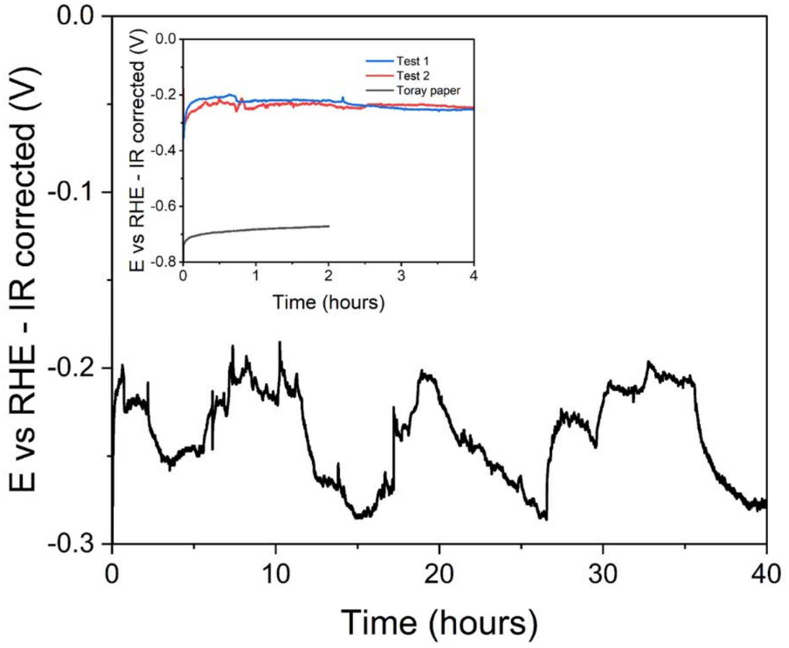

Figure S22: 40-hours long chronopotentiometric measurement at -10 mA cm_{geo}^{-2}. In the inset, repetitive 4 hours-long CP tests.

Table S8: Key electrochemical HER parameters derived from linear sweep voltammetry (E_{Onset}) and chronopotentiometric scans (η^{HER}) for NiAs, alkaline pH. All potentials are negative, minus sign omitted.

	E_{Onset}^{HER} [a]	$\eta_{-10mA\ cm^{-2}\ geo\ (t=2h)}^{HER}$
	mV	
NiAs	≈ 371	≈ 225 (200-300[b])
Toray paper	≈ 753	≈ 670

[a] *E_{Onset} defined using the tangent method on the two slopes obtained by LSV*

[b] *Data collected after 35-40 hours of operation*

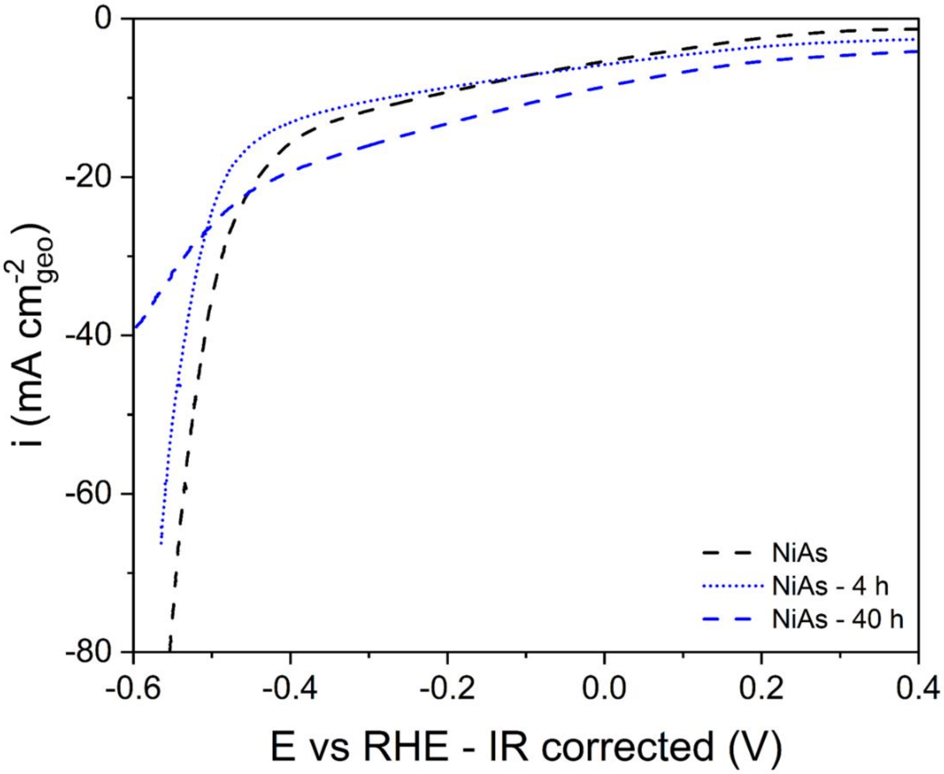

Figure S23: Linear sweep voltammetries (scan rate = 2 mV s^{-1}) collected on pristine (solid line) and used (dashed line) NiAs/Toray paper electrodes after CPs in 1 M KOH

LSV curves, registered for both the bare support and NiAs/Toray paper electrodes (Figure S21a), evidence the HER activity of the material under alkaline conditions. Indeed, E_{Onset} shifts from *ca.* -0.750 V vs RHE to *ca.* -0.370 V vs RHE upon depositing NiAs onto the carbon-based support. Differently from what observed under acidic environment, LSVs variability is more relevant in this case (Figure S21b), introducing a larger error on the determination of E_{Onset} (*ca.* ± 40 mV). Figure S21c also reports the ECSA-corrected LSV. To the best of our knowledge, no TMAs-based HER catalysts operating under alkaline

environment have been reported in the literature; therefore, no direct comparison with similar samples are possible.

As previously observed for CVs, a double slope is observed in the LSVs as well. Interestingly, the presence of a first, milder slope is detected also on the bare support (blue curve in Figure S21a), corroborating the hypothesis of a pre-HER support reduction. Anyway, the marked increase of this initial slope upon NiAs deposition (black curve in Figure S21a) opens to the possibility of this phenomenon to be also correlated with HER onto NiAs. Being this a complex matter of investigation and being outside from the main objectives of this paper (which focuses on acidic HER on TMAs as possible replacement of PGM-based electrocatalysts), further dedicated studies will be needed to elucidate this phenomenon.

The 4-hours long CP (Figure 21d, average of two replicates, check Figure S22 inset), run at -10 mA cm^{-2} (geometric current), stabilizes around $\eta^{HER}_{-10mA\ cm^{-2}\ geo}$ of 250-300 mV (Table S8). Regarding the long time stability (Figure S22), the outcome of a 40 hours long CP (always run at -10 mA cm^{-2} geometric current) is consistent with what observed at short scale: the overpotential indeed oscillates between 200 and 300 mV. The large oscillations reported in Figure S22 are most likely imputable to day-night temperature variations. As a matter of fact, the electrochemical cell used for the stability test has not been thermally insulated and overpotential fluctuactions come in repetitive "waves" with a *ca.* 24 hours period. The $\eta^{HER}_{-10mA\ cm^{-2}\ geo}$ exhibited by NiAs under alkaline conditions is by all means more promising than that recorded under acidic

conditions. However, the $\eta^{HER}_{-10mA\,cm^{-2}\,geo}$ here reported is still far from recent state-of-the-art electrocatalysts for alkaline HER [11–14], although several of them have the main drawback of being PGM-based.

Post-CP LSVs (Figure S23) indicate an almost full retention of the HER activity of NiAs after short operation times (i.e. 4 hours, blue dotted line in Figure S23), while a loss of activity, represented by the decreased i vs E slope after the reaction onset, is detected after longer CPs (i.e. 40 hours, blue dashed line in Figure S23). It is worth noticing that the pre-HER onset slope is still present after long time continuous operation at cathodic currents, further suggesting that this first cathodic phenomenon might be something more complex than the simple support reduction.

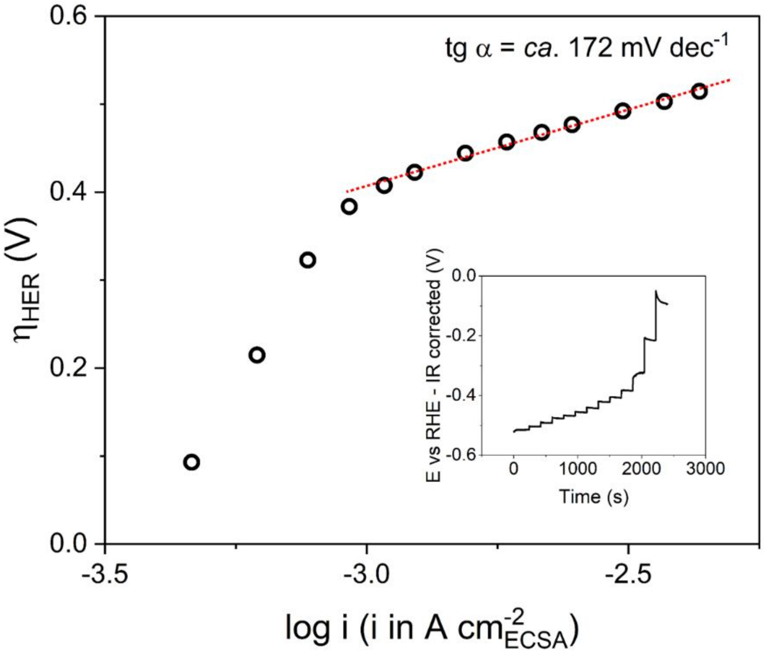

Figure S24: Tafel slope extracted from chronopotentiometric test. The electrochemical trace from which overpotential versus log i data have been obtained is reported in the inset

Going to NiAs microkinetics, the catalyst Tafel slope has been measured according to the above-discussed procedure. Figure S24, as expected, features two regions with different slopes, due to the simultaneous reactions (allegedly, surface reduction of the carbon-based support and actual HER on NiAs). The high current region is the one containing the information related to HER kinetics on NiAs under alkaline conditions. However, the contribution of the support reduction (or, more in general, of the first cathodic phenomenon) cannot be ruled out in these tests. The Tafel slope measured for the sample in 1 M KOH is again > 120 mV dec^{-1}. The reasons why this might happen have been thoroughly discussed in the previous section. We should also point out that the presence of a parallel faradaic process forces the present Tafel slope to be regressed from a high current region; considering the cell geometry and the absence of forced

convection (i.e. no RDE testing have been carried out), diffusional problems might arise at such high currents, hampering a correct determination of kinetic parameters.

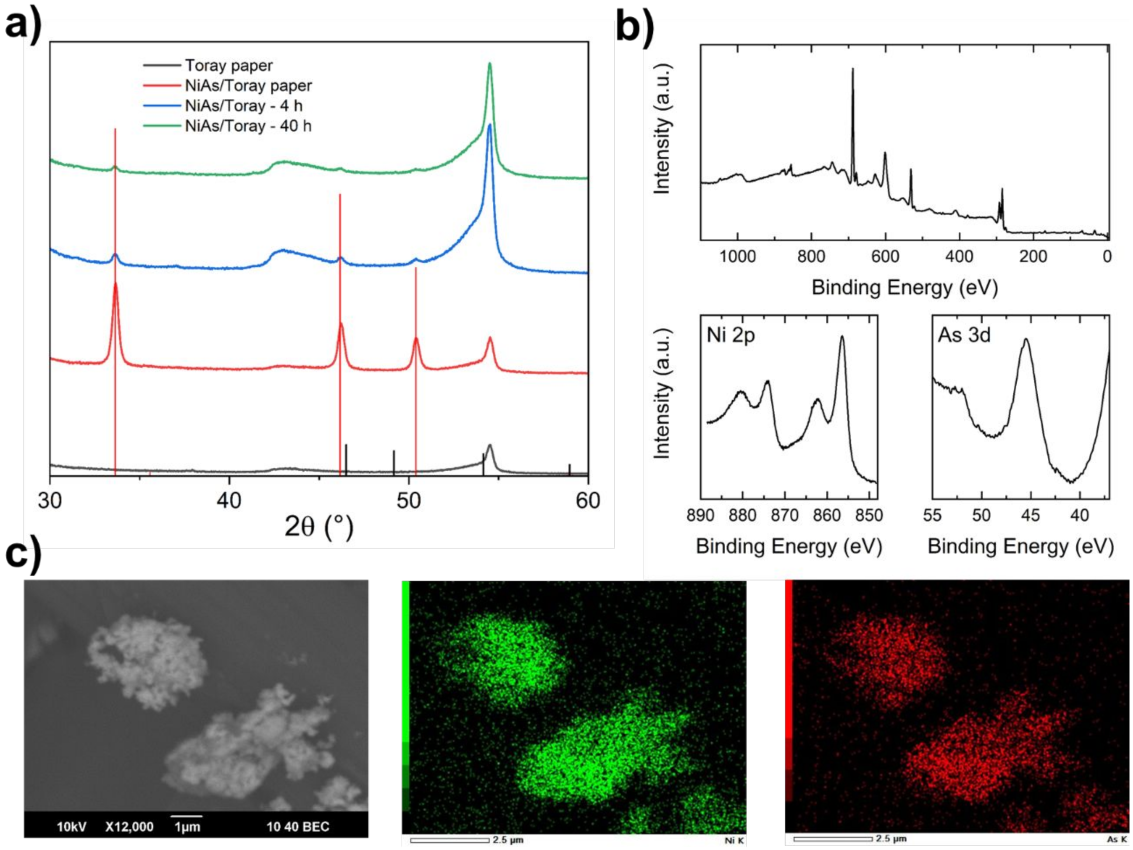

Figure S25: (a) XRD patterns, (b) XPS survey (top) and HR-XPS of Ni 2p and As 3d regions and (c) SEM images with related Ni and As elemental maps of NiAs/Toray paper electrodes after long CP scans under alkaline HER conditions.

A complete physical-chemical characterization has been carried out on the electrodes after long CP scans. XRD patterns (Figure S25a) show that the phase purity of the sample is retained (NiAs reflections still present, no new peaks detected), although a non-negligible decrease in peaks' intensity can be noticed. This phenomenon might indicate either dissolution of NiAs or amorphization. XPS spectra (Figure S25b) depict a Ni-enriched surface with Ni mainly being in the +2 oxidation state, suggesting the presence of $Ni(OH)_2$

and/or NiOOH. On the other hand, SEM/EDS elemental maps and the related quantification are consistent with NiAs phase retention (Ni to As ratio being *ca.* 1:1, precisely 52:48). The full picture obtained from these characterizations indicates surely an improved stability of NiAs under alkaline HER conditions with respect with acidic ones. Anyway, the surface enrichment in Ni, revealed by XPS, points toward a Ni oxy/hydroxide active phase being formed upon operative conditions, similarly to what has been observed under alkaline OER conditions (refer to the "Oxygen Evolution Reaction (OER)" section in the main text and, in particular, to Figure 5).

Overall, the NiAs HER performance under alkaline conditions definitely outpaces the one registered at acidic pH, both in terms of electrocatalytic metrics and stability. Despite that, we decided to focus on the acidic environment for a deeper investigation as replacement of Pt (and PGM-based in general) at the cathode of Proton Exchange Membrane (PEM) electrolyzers is the main aim of TM compounds HER testing. On the other hand, although alkaline HER can already rely on relatively efficient and especially very cheap cathodes (e.g. perforated Ni-coated stainless steel electrode, [15]), a further investigation of TMAs HER performance at alkaline pH is undoubtedly of interest.

1.5 Alkaline OER: electrochemical data

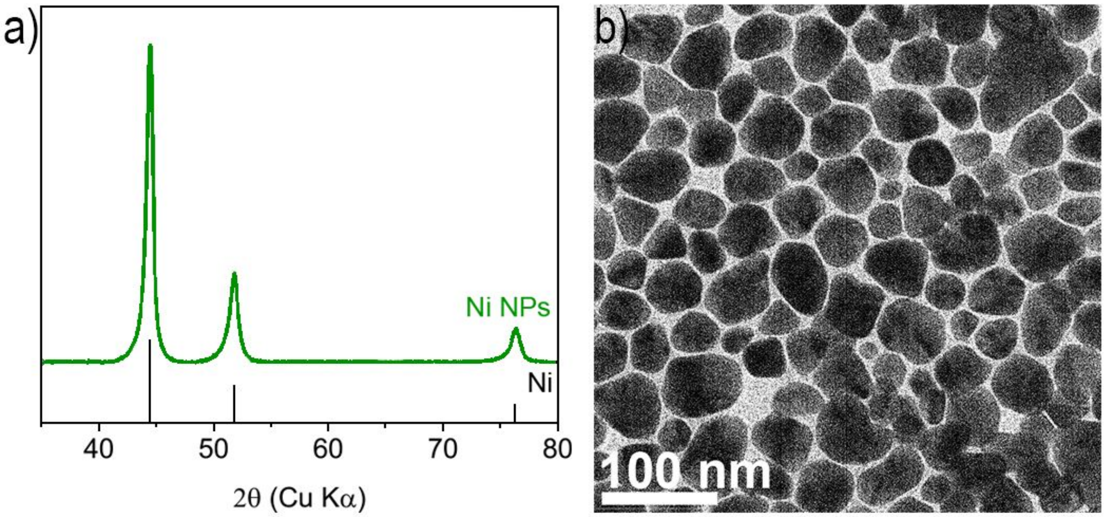

Figure S26: (a) XRD pattern and (b) BF-TEM image of Ni^0 NCs

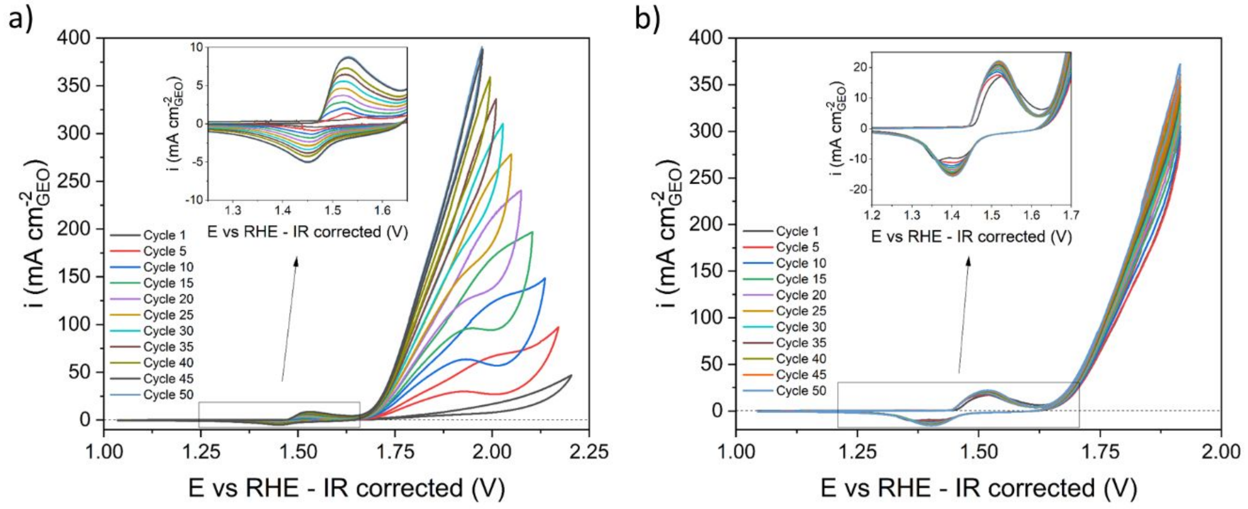

Figure S27: Sample conditioning cyclovoltammetric curves (scan rate = 100 mV s^{-1}) ran on (a) NiAs/Toray paper and (b) Ni^0/Toray paper electrodes. Insets: magnification of the region of Ni(II) to Ni(III) oxidation (and viceversa on the backward scans).

<u>Additional comments on the CVs shapes and peaks</u>

CVs collected on NiAs/Toray paper electrodes show a peculiar shape, characterized by the presence of a faradaic peak at large anodic potentials (*ca*. 2 V vs RHE) that disappears upon potential cycling. As multiple cycles are recorded, the OER performance of NiAs NCs increases steadily, reaching a stable behavior between cycles 45 to 50. The disappearance of the peak (most likely related to the As leaching through oxidation and related surface reconstruction) and the increase of performances indicate that NiAs is actually the pre-catalysts, transforming during cycling to the real active phase,[16] most likely Ni oxy/hydroxides, as further confirmed by the post-catalysis characterizations (Figure 5 in the main text).

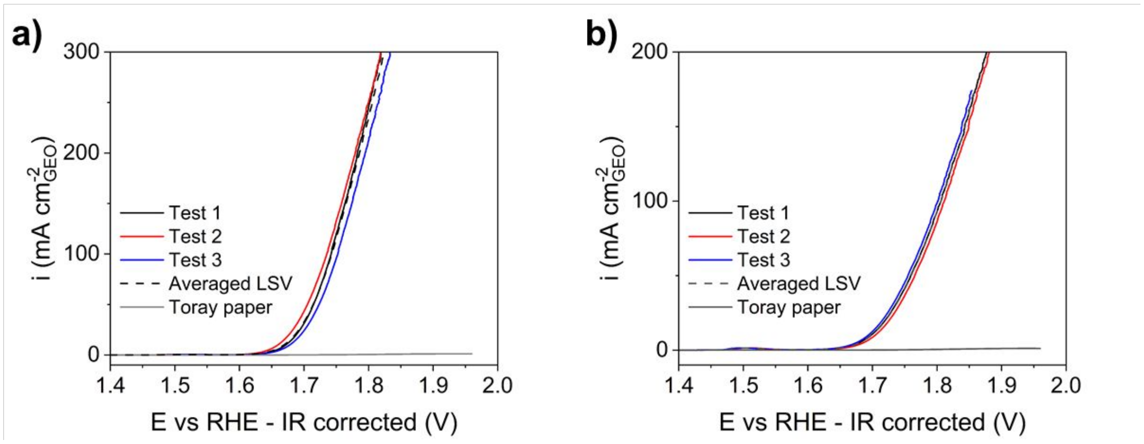

Figure S28: Replicated LSV curves (scan rate = 2 mV s^{-1}) registered on three independent (a) NiAs/Toray paper electrodes and (b) Ni^0/Toray paper in comparison with the bare support.

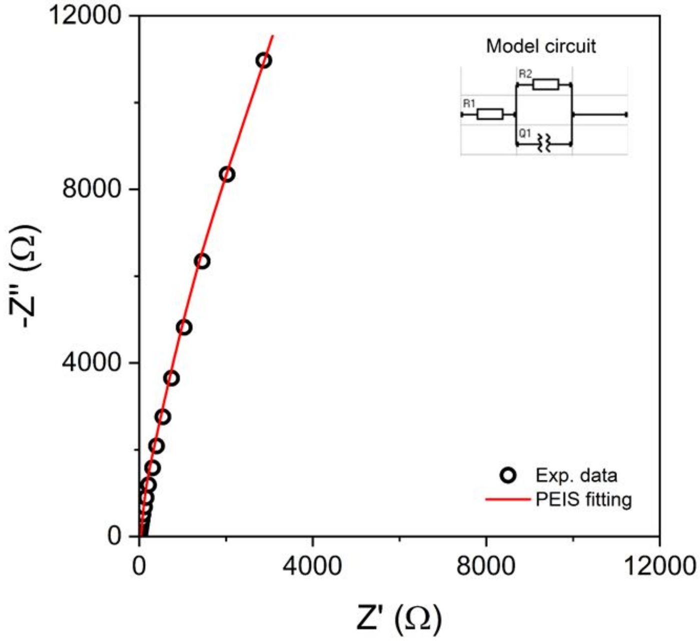

Figure S29: Example of PEIS experimental data and fitting (according to the model presented in the inset). PEIS collected in non-faradaic region, details available in Table S5

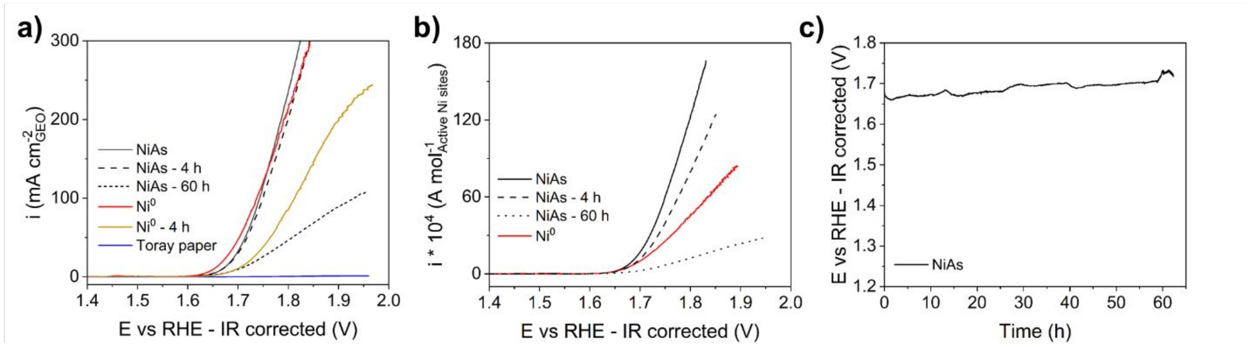

Figure S30: Evaluation of the electrochemical OER activity of NiAs. Linear sweep voltammetries (scan rate = 2 mV s^{-1}) of NiAs and Ni^0 NCs displayed as potential vs (a) geometrical and (b) number of active sites-normalized current densities. (c) 4 hours long chronopotentiometric measurement at 10 mA cm_{geo}^{-2}. This figure mirrors Figure 4 in the main text but displays the behavior of NiAs and Ni^0 NCs at (a, b) larger current densities and (c) longer times.

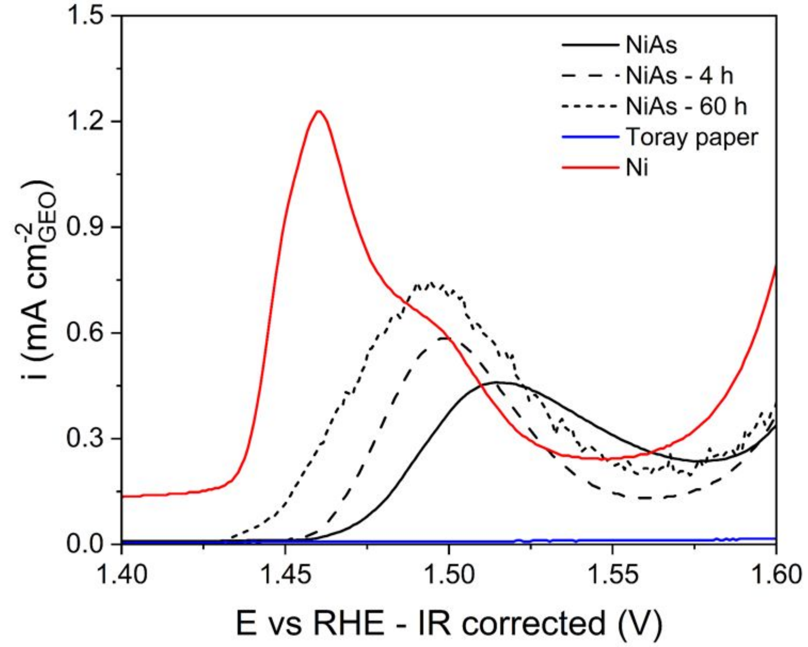

Figure S31: Ni(II) to Ni(III) oxidation peaks registered by LSV (details available in Table S1) on NiAs NCs, Ni^0 NCs and the bare support

Additional comments on the Ni(II) to Ni(III) oxidation peaks

As is clear from the electrochemical traces reported in Figure S31, the formation of the Ni(III) active site is influenced, in terms of formation energy barrier (i.e. the actual potential of Ni(III) oxidation) by the nature of the Ni-base catalyst. Ni^0 NCs display an oxidation peak centered at *ca*. 1.45 V vs RHE, consistent with the literature.[17] On the other hand, Ni(II) to Ni(III) oxidation appears to be hindered on NiAs, presenting a slightly larger peak potential (*ca*. 1.52 V vs RHE, black line in Figure S31), shifting to lower values (i.e. closer to Ni^0 NCs) upon operation. The higher energy barrier for Ni(III) formation in NiAs is in contrast with what reported by Masa et al.,[17] in which a shift of the oxidation peak to less anodic potentials was detected and attributed to the electronegativity of As ($E.N._{As} = 2.18$, $E.N._{Ni} = 1.91$). However, it has to be stressed that Masa et al. investigated Ni-metalloids alloys, not proper nickel arsenide. Considering the post-catalysis characterization results (Figure 5 in the main text), it can be concluded that Ni(III) formation in the Ni oxy/hydroxides shell grown on the surface of NiAs NCs during OER testing is influenced by the NiAs core itself; such effect seems to become less prominent as the Ni oxy/hydroxides shell grows, gradually shifting the Ni(III) oxidation peak to lower potentials (approaching Ni^0 NCs one).

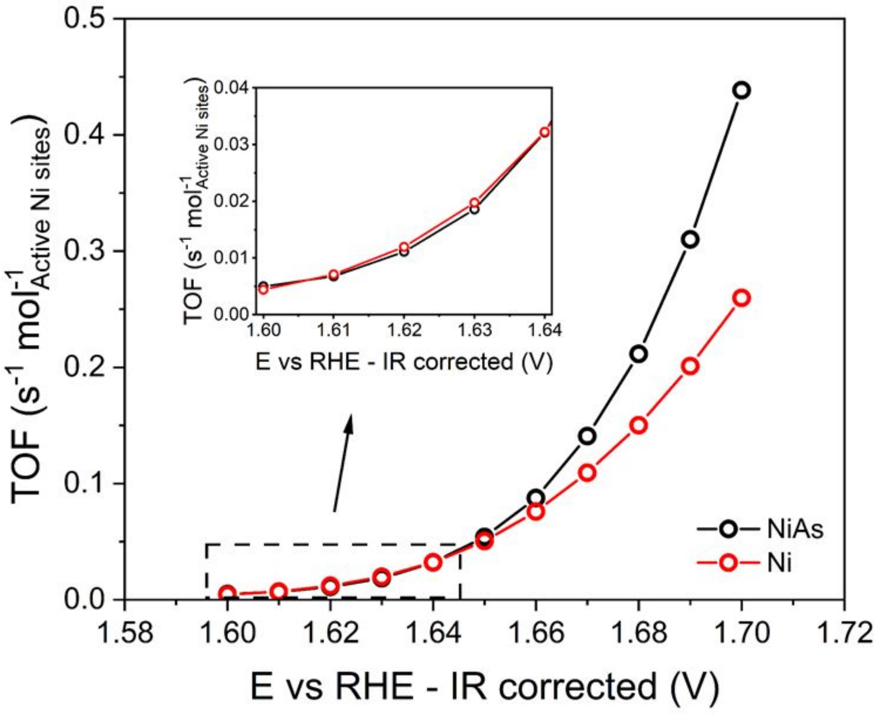

Figure S32: TOF calculated at various potentials for NiAs NCs and Ni^0 NCs. Details of TOF calculations are available in the dedicated section.

Table S9: Current density and related TOF calculated at different potential. Input data (E, i) from LSV curves reported in the main text, Figure 4a.

E vs RHE - IR corrected	NiAs		Ni^0	
	i	TOF	i	TOF
V	*mA cm^{-2}$_{Geo}$*	*s^{-1} mol$_{Active\ Ni\ sites}$$^{-1}$*	*mA cm^{-2}$_{Geo}$*	*s^{-1} mol$_{Active\ Ni\ sites}$$^{-1}$*
**1.6**	0.371	0.00499	0.801	0.0044
**1.61**	0.503	0.00676	1.293	0.0071
**1.62**	0.826	0.0111	2.175	0.01195
**1.63**	1.381	0.01857	3.593	0.01974
**1.64**	2.389	0.03212	5.868	0.03224
**1.65**	4.014	0.05396	9.198	0.05054
**1.66**	6.512	0.08754	13.818	0.07593
**1.67**	10.471	0.14077	19.900	0.10935
**1.68**	15.74	0.2116	27.338	0.15022
**1.69**	23.065	0.31007	36.583	0.20102
**1.7**	32.615	0.43846	47.265	0.25971

Turnover frequency (TOF) calculation

TOF is a useful electrochemical key parameter that displays the electrocatalytic activity of a material as the amount of product formed per unit time per given amount of catalyst [4]. Among the numerous equations used to determine the TOF in electrocatalysis, in this paper the following one has been chosen:

$$TOF = \frac{i}{x \times F \times n}$$

in which i is the current density (A cm^{-2}_{Geo}), x is the number of active sites ($mol_{Active\ Ni\ sites}$ cm^{-2}_{Geo}), F is the Faraday constant (96485 C mol_e^{-1}) and n the number of electrons transferred to generate one single molecule of product (4 mol_e^{-1} per O_2 molecule in the case of OER). With *x* being determined by integration of the Ni(II) to Ni(III) oxidation peak of Ni-based electrodes, TOF (s^{-1} $mol_{Active\ Ni\ sites}^{-1}$) is generally reported as a function of the applied potential (i.e. retrieving i data from LSV curves at fixed E), as displayed in Figure S32.

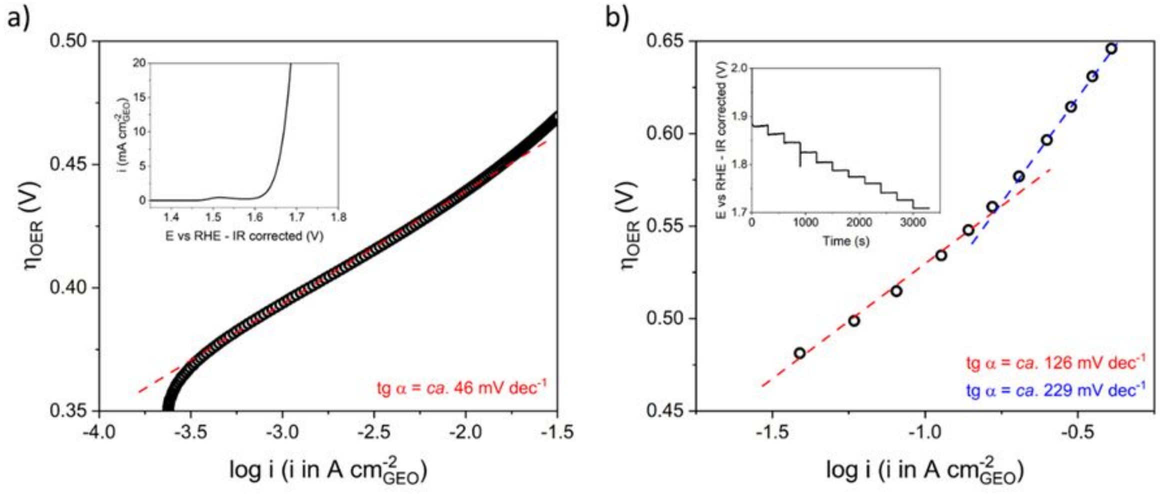

Figure S33: Tafel slopes of NiAs NCs as extracted from (a) linear sweep voltammetry and (b) chronopotentiometric tests. Electrochemical traces from which overpotential versus log i data have been obtained are reported in the insets.

<u>Extended discussion on OER Tafel slopes determination and significance</u>

In terms of kinetic parameters, literature reports OER Tafel slopes for Ni-based catalysts ranging from 50-60 to 120 mV dec^{-1} [6]. On the other hand, Masa et al. obtained a Tafel slope of *ca.* 59 mV dec^{-1} for NiAs [17], although the authors do not clearly indicate whether the regression has been performed on potentiostatically or (most likely) potentiodynamically-collected data. For the sake of completeness, both approaches have been followed in the present paper. When regressing the NiAs Tafel slope from data collected by linear sweep voltammetry, a value of *ca.* 46 mV dec^{-1} is obtained (Figure S33a); such slope is consistent with those reported by Masa et al. for different Ni-metalloid alloys.[17] A more rigorous determination of the Tafel slope by a potentiostatic method (Figure S33b, details available in the dedicated section) highlights instead a higher slope and its dependence on the overpotential, as often reported for OER catalysts.[18–20] For $\eta <$ 0.55 V, NiAs exhibits a Tafel slope of *ca.* 120 mV dec^{-1}; according to microkinetic analyses and theoretical models, such slope value is observed for large coverages of surface species formed in the step previous to

the rate-determining one.[6] Therefore, the single Tafel slope is in this case not sufficient to unveil the exact step representing the bottleneck in the OER mechanism onto NiAs. In the high overpotential region (η > 0.55 V), a steeper value of the Tafel slope is obtained (*ca.* 225 mV dec^{-1}). Although already documented for Pt in 1 M KOH [20], the almost doubling of the Tafel slope with increasing potential is difficult to be addressed as it could stem from both surface coverage-related issue or simply from diffusional limitations, with the latter likely occurring when achieving large current densities under a typical three-electrode configuration.

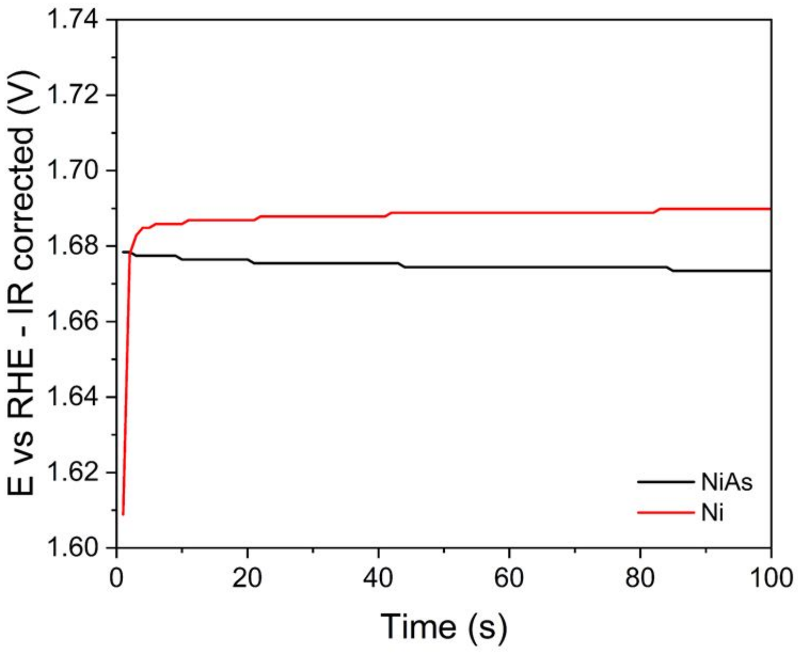

Figure S34: First 100 seconds of chronopotentiometric scans on NiAs NCs and Ni^0 NCs electrodes. I = 2.5 mA, equal to i = 10 mA cm_{geo}^{-2}.

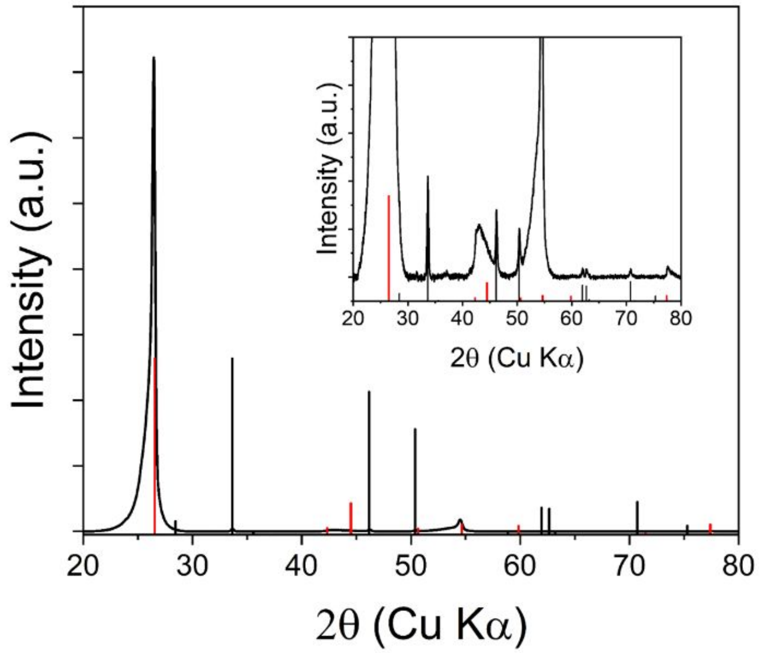

Figure S35: XRD pattern collected on NiAs/Toray paper electrodes after 60 hours of OER testing (chronopotentiometric scan, 10 mA cm_{geo}^{-2}). Red and black lines are the reference reflections of graphite (ICSD 76767) and NiAs (hexagonal, ICSD 611040), respectively.

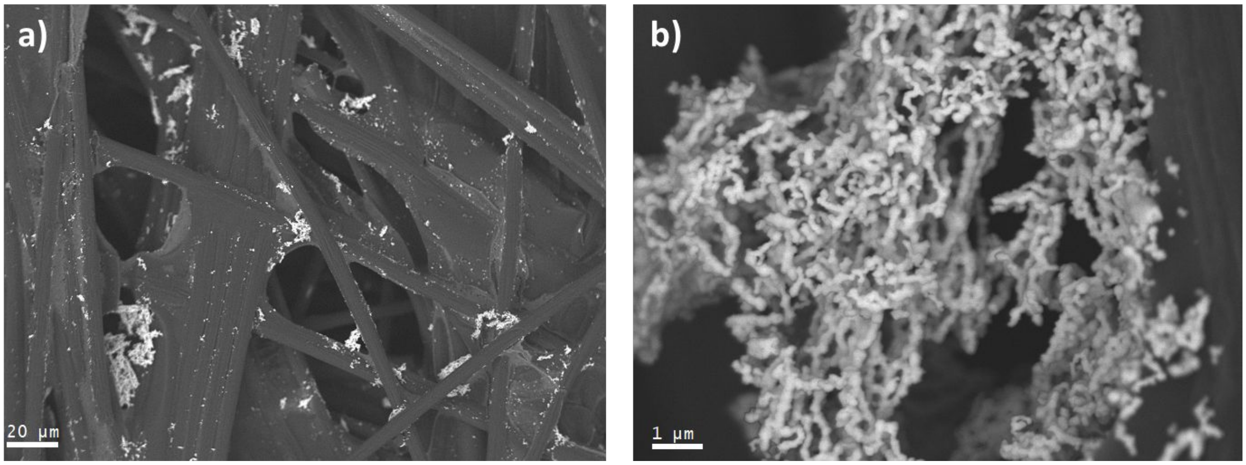

Figure S36: (a) Low and (b) high magnification FE-SEM images collected on NiAs/Toray paper electrodes after 60 hours-long chronopotentiometric test at 10 mA cm_{geo}^{-2}.

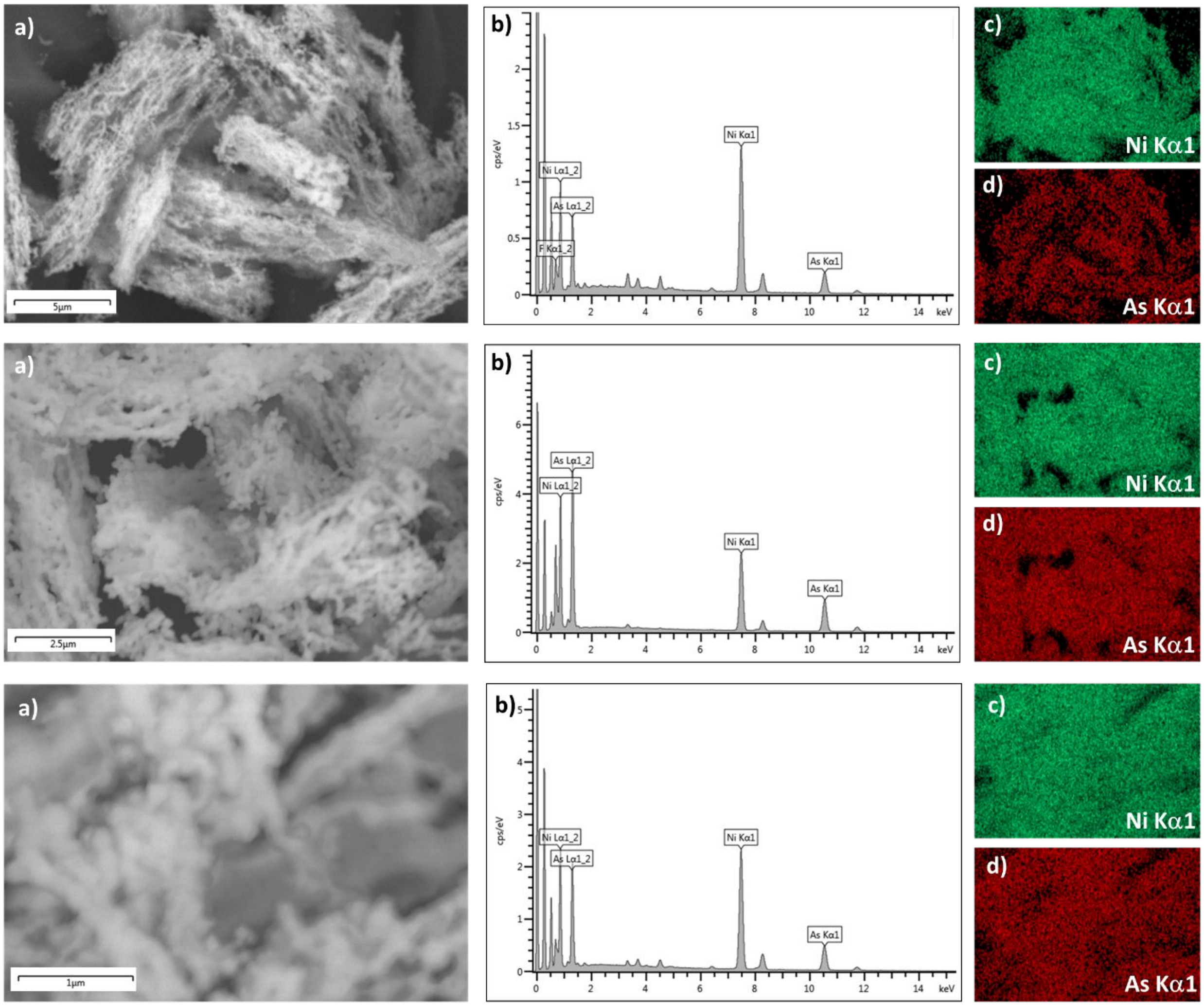

Figure S37: (a) FE-SEM images and (b) corresponding SEM-EDS spectra and (c,d) elemental maps collected on NiAs/Toray paper electrodes after 60 hours-long chronopotentiometric test at 10 mA cm_{geo}^{-2}

Table S10: Atomic percentage of Ni and As from EDS analyses reported in Figure S37

	**Atomic % by SEM-EDS**	
	Ni-Kα	As-Kα
**Top**	71.92	28.02
**Middle**	67.53	32.47
**Bottom**	70.79	29.21
**Average**	70.08	29.92

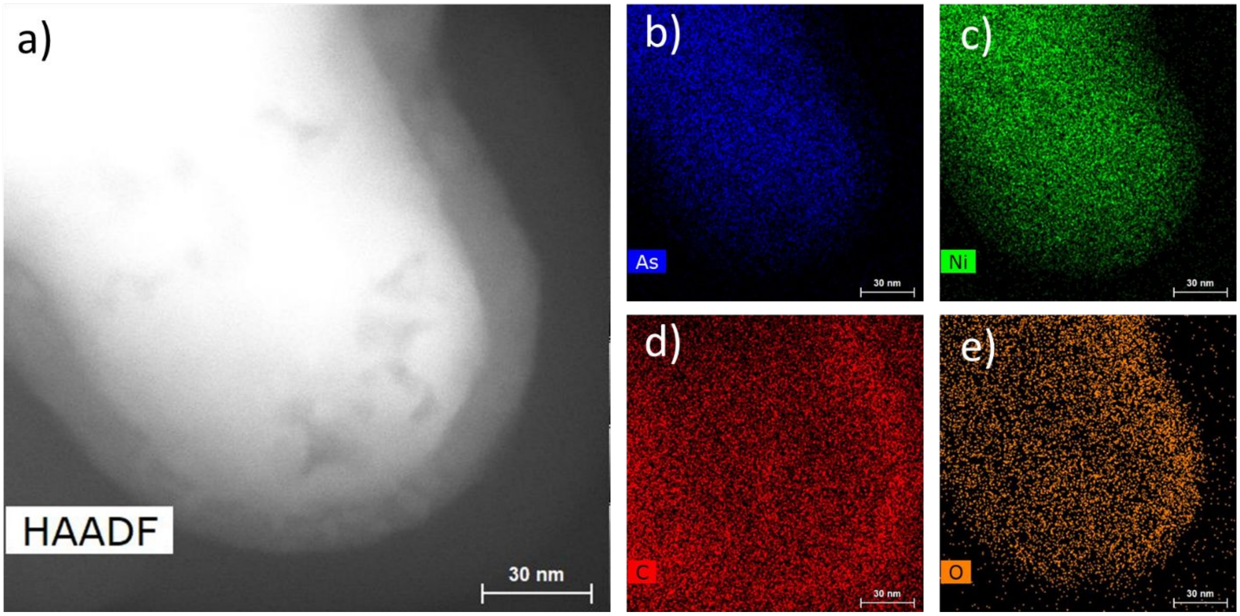

Figure S38: (a) Additional HAADF-STEM image of NiAs NCs detached from the support after OER chronoamperometric tests and related (b) arsenic, (c) nickel, (d) carbon and (e) oxygen STEM-EDS elemental maps.

Additional references

(1) Morales, D. M.; Risch, M. Seven Steps to Reliable Cyclic Voltammetry Measurements for the Determination of Double Layer Capacitance. *JPhys Energy* **2021**, *3* (3). https://doi.org/10.1088/2515-7655/abee33.

(2) Anantharaj, S.; Noda, S.; Driess, M.; Menezes, P. W. The Pitfalls of Using Potentiodynamic Polarization Curves for Tafel Analysis in Electrocatalytic Water Splitting. *ACS Energy Lett.* **2021**, *6*, 1607–1611. https://doi.org/10.1021/acsenergylett.1c00608.

(3) Gauthier, J. A.; King, L. A.; Stults, F. T.; Flores, R. A.; Kibsgaard, J.; Regmi, Y. N.; Chan, K.; Jaramillo, T. F. Transition Metal Arsenide Catalysts for the Hydrogen Evolution Reaction. *J. Phys. Chem. C* **2019**, *123* (39). https://doi.org/10.1021/acs.jpcc.9b05738.

(4) Anantharaj, S.; Karthik, P. E.; Noda, S. The Significance of Properly Reporting Turnover Frequency in Electrocatalysis Research. *Angew. Chemie - Int. Ed.* **2021**, *60* (43), 23051–23067. https://doi.org/10.1002/anie.202110352.

(5) Trasatti, S.; Petrii, O. A. International Union of Pure and Applied Chemistry Physical Chemistry Division Commission on Electrochemistry: Real Surface Area Measurements in Electrochemistry. *Pure Appl. Chem.* **1991**, *63* (5), 711–734. https://doi.org/10.1351/pac199163050711.

(6) Shinagawa, T.; Garcia-Esparza, A. T.; Takanabe, K. Insight on Tafel Slopes from a Microkinetic Analysis of Aqueous Electrocatalysis for Energy Conversion. *Sci. Rep.* **2015**, *5* (August), 1–21. https://doi.org/10.1038/srep13801.

(7) Fletcher, S. Tafel Slopes from First Principles. *J. Solid State Electrochem.* **2009**, *13* (4), 537–549. https://doi.org/10.1007/s10008-008-0670-8.

(8) Marković, N. M.; Grgur, B. N.; Ross, P. N. Temperature-Dependent Hydrogen Electrochemistry on Platinum Low-Index Single-Crystal Surfaces in Acid Solutions. *J. Phys. Chem. B* **1997**, *101* (27), 5405–5413. https://doi.org/10.1021/jp970930d.

(9) Markovića, N. M.; Sarraf, S. T.; Gasteiger, H. A.; Ross, P. N. Hydrogen Electrochemistry on Platinum Low-Index Single-Crystal Surfaces in Alkaline Solution. *J. Chem. Soc., Faraday Trans.* **1996**, *92* (20), 3719–3725. https://doi.org/10.1039/FT9969203719.

(10) Fang, Y. H.; Liu, Z. P. Tafel Kinetics of Electrocatalytic Reactions: From Experiment to First-Principles. *ACS Catal.* **2014**, *4* (12), 4364–4376. https://doi.org/10.1021/cs501312v.

(11) Zhai, P.; Xia, M.; Wu, Y.; Zhang, G.; Gao, J.; Zhang, B.; Cao, S.; Zhang, Y.; Li, Z.; Fan, Z.; Wang, C.; Zhang, X.; Miller, J. T.; Sun, L.; Hou, J. Engineering Single-Atomic Ruthenium Catalytic Sites on Defective Nickel-Iron Layered Double Hydroxide for Overall Water Splitting. *Nat. Commun.* **2021**, *12* (1), 1–11. https://doi.org/10.1038/s41467-021-24828-9.

(12) Xu, Y.; Yu, S.; Ren, T.; Liu, S.; Wang, Z.; Li, X.; Wang, L.; Wang, H. Hydrophilic/Aerophobic Hydrogen-Evolving Electrode: NiRu-Based Metal-Organic Framework Nanosheets in Situ Grown on Conductive Substrates. *ACS Appl. Mater. Interfaces* **2020**, *12* (31), 34728–34735. https://doi.org/10.1021/acsami.0c03333.

(13) Zhang, Z.; Liu, G.; Cui, X.; Chen, B.; Zhu, Y.; Gong, Y.; Saleem, F.; Xi, S.; Du, Y.; Borgna, A.; Lai, Z.; Zhang, Q.; Li, B.; Zong, Y.; Han, Y.; Gu, L.; Zhang, H. Crystal Phase and Architecture Engineering of Lotus-Thalamus-Shaped Pt-Ni Anisotropic Superstructures for Highly Efficient Electrochemical Hydrogen Evolution. *Adv. Mater.* **2018**, *30* (30). https://doi.org/10.1002/adma.201801741.

(14) Mahmood, J.; Li, F.; Jung, S. M.; Okyay, M. S.; Ahmad, I.; Kim, S. J.; Park, N.; Jeong, H. Y.; Baek, J. B. An Efficient and PH-Universal Ruthenium-Based Catalyst for the Hydrogen Evolution Reaction. *Nat. Nanotechnol.* **2017**, *12* (5), 441–446. https://doi.org/10.1038/nnano.2016.304.

(15) IRENA. *Green Hydrogen Cost Reduction: Scaling up Electrolysers to Meet the 1.5^{0}C Climate Goal*; Abu Dhabi, 2020.

(16) Anantharaj, S.; Noda, S. Nickel Selenides as Pre-Catalysts for Electrochemical Oxygen Evolution Reaction: A Review. *Int. J. Hydrogen Energy* **2020**, *45* (32), 15763–15784. https://doi.org/10.1016/j.ijhydene.2020.04.073.

(17) Masa, J.; Piontek, S.; Wilde, P.; Antoni, H.; Eckhard, T.; Chen, Y. T.; Muhler, M.; Apfel, U. P.; Schuhmann, W. Ni-Metalloid (B, Si, P, As, and Te) Alloys as Water Oxidation Electrocatalysts. *Adv. Energy Mater.* **2019**, *9* (26), 1–8. https://doi.org/10.1002/aenm.201900796.

(18) Smith, R. D. L.; Prévot, M. S.; Fagan, R. D.; Zhang, Z.; Sedach, P. A.; Siu, M. K. J.; Trudel, S.; Berlinguette, C. P. Photochemical Route for Accessing Amorphous Metal Oxide Materials for Water Oxidation Catalysis. *Science (80-.).* **2013**, *340* (6128), 60–63. https://doi.org/10.1126/science.1233638.

(19) Shi, H.; Zhao, G. Water Oxidation on Spinel NiCo 2 O 4 Nanoneedles Anode: Microstructures, Specific Surface Character, and the Enhanced Electrocatalytic Performance. *J. Phys. Chem. C* **2014**, *118* (45), 25939–25946. https://doi.org/10.1021/jp508977j.

(20) Damjanovic, A.; Dey, A.; Bockris, J. O. Kinetics of Oxygen Evolution and Dissolution on Platinum Electrodes. *Electrochim. Acta* **1966**, *11* (7), 791–814. https://doi.org/10.1016/0013-4686(66)87056-1.
